# Efficacy of Ultrasound-Guided Pectointercostal Fascial Block Versus Control and Alternative Regional Techniques in Cardiac Surgery: A Systematic Review and Meta-Analysis with Dose–Response Analysis

**DOI:** 10.3390/medicina62101890

**Published:** 2026-09-29

**Authors:** Mahmoud Elnahas, Mostafa Zidan, Yousuf Abusamra, Mohamed Hassan, Babak Saravi, Sieglinde Hochrein, Philip Lang

**Affiliations:** 1 Department of Anesthesiology, Intensive Care Medicine, and Pain Therapy, Sozialstiftung Bamberg, 96049 Bamberg, Germany; mahmoud.elnahas@web.de (M.E.); philip.lang@sozialstiftung-bamberg.de (P.L.); 2 Department of Anaesthesia, Cambridge University Hospitals NHS Foundation Trust, Cambridge CB2 0QQ, UK; mostafa.zidan2@nhs.net; 3 Department of Anaesthesia, Manchester University NHS Foundation Trust, Manchester M13 9WL, UK; yousuf.abusamra@mft.nhs.uk; 4 Department of Thoracic and Cardiovascular Surgery, Julius-Maximilians-University Hospital, 97080 Wuerzburg, Germany; hassan_m@ukw.de; 5 Department of Oral, Maxillofacial and Facial Plastic Surgery, Medical Faculty and University Hospital Düsseldorf, Heinrich-Heine-University Düsseldorf, 40225 Düsseldorf, Germany

**Keywords:** pectointercostal fascial block, cardiac surgery, sternotomy, regional anesthesia, opioid consumption, meta-analysis, GRADE

## Abstract

*Background and Objectives*: Ultrasound-guided pectointercostal fascial block (USG-PIFB) is increasingly used for post-sternotomy analgesia, but its efficacy remains uncertain. Prior meta-analyses pooled ultrasound-guided blocks with surgeon-performed infiltration, introducing heterogeneity. We aimed to determine the analgesic efficacy of USG-PIFB in adult cardiac surgery by restricting inclusion to ultrasound-guided techniques. *Materials and Methods*: Six databases were searched through December 2025, with an update search of open sources and trial registries in September 2026, for randomized controlled trials comparing USG-PIFB with control (Comparison 1) or with an alternative regional technique (Comparison 2) in adults undergoing sternotomy. The primary outcome was 24-h opioid consumption in intravenous morphine milligram equivalents. Random-effects meta-analysis (REML, Knapp–Hartung), subgroup, sensitivity and meta-regression analyses, an exploratory trial sequential analysis and GRADE assessment were performed. *Results*: Twenty-nine trials (2664 participants) were included: 24 in Comparison 1 and eight in Comparison 2. In 13 trials (902 participants), USG-PIFB reduced 24-h opioid consumption (mean difference −6.60 mg; 95% CI, −10.92 to −2.27; I^2^ = 97.7%), but the 95% prediction interval (−22.24 to 9.04 mg) included no effect and small-study effects could not be excluded. Pain scores were lower by 0.8 to 1.4 points at rest (extubation, 12 and 24 h) and during movement (12 h). Extubation was earlier and intensive care stay shorter, with extreme heterogeneity. No dose–response relationship was detected, and the difference from deep parasternal techniques was near zero. No local anesthetic systemic toxicity or block-attributable pneumothorax was reported; one block-site hematoma occurred in a USG-PIFB recipient. Certainty of evidence was low for rescue analgesic use and block-related complications and very low for all other outcomes. *Conclusions*: USG-PIFB may modestly reduce 24-h opioid consumption, but the magnitude and reproducibility of this effect across clinical settings remain uncertain. It is best regarded as one component of multimodal analgesia, and adequately powered multicentre trials are needed.

## 1. Introduction

Cardiac surgery via median sternotomy remains among the most painful surgical procedures, with moderate-to-severe acute pain reported by more than half of patients during the first 48 postoperative hours [1,2]. The sternotomy incision disrupts osseous, periosteal, and intercostal soft-tissue structures innervated by the anterior cutaneous branches of the T2–T6 intercostal nerves, producing a nociceptive burden that is often undertreated [3,4,5]. Inadequately controlled post-sternotomy pain impairs respiratory mechanics—particularly the capacity for deep inspiration and effective cough—and has been consistently associated with atelectasis, pneumonia, prolonged mechanical ventilation, and delayed functional recovery [1]. Pain-related inhibition of early mobilization may additionally contribute to thromboembolic complications and delirium, compounding the morbidity of the index procedure [6,7]. Effective perioperative analgesia is therefore a prerequisite not only for patient comfort but for the broader goals of enhanced recovery after cardiac surgery, including early extubation, timely physiotherapy, and reduced intensive care and hospital lengths of stay [6,7].

Post-sternotomy pain management has traditionally relied on systemic opioids administered via intravenous patient-controlled analgesia. While effective, opioid-based strategies carry dose-dependent risks of respiratory depression, sedation, postoperative ileus, delirium, and prolonged mechanical ventilation—effects that are particularly consequential in the hemodynamically vulnerable cardiac surgical population [6,7]. Neuraxial techniques, including thoracic epidural analgesia and paravertebral blockade, can provide excellent sternal analgesia, but their application in cardiac surgery is constrained by the obligatory systemic anticoagulation for cardiopulmonary bypass, which elevates the risk of epidural hematoma to an extent that most practitioners consider unacceptable [4]. These logistical and safety barriers have stimulated interest in peripheral fascial plane blocks of the anterior chest wall that can be performed safely in fully anticoagulated patients [5,8].

The superficial parasternal intercostal plane block—also referred to as the pecto-intercostal fascial block (PIFB) or parasternal intercostal block (PIB)—is an ultrasound-guided interfascial injection technique in which local anesthetic is deposited between the pectoralis major and external intercostal muscles at the level of the costal cartilages, adjacent to the sternal border [2,5]. First described by de la Torre and colleagues for breast surgery analgesia [9], this technique was subsequently adopted for median sternotomy on the basis of its anatomical rationale: the anterior cutaneous branches of the T2–T6 intercostal nerves traverse this fascial plane en route to the skin and periosteum of the sternum [4,5]. The block is technically straightforward, can be performed bilaterally at multiple intercostal levels in either anesthetized or awake patients, and carries a low risk of pneumothorax because the needle trajectory is superficial to the internal intercostal membrane [2]. Both single-shot and continuous catheter-based delivery have been described, with the latter permitting extended analgesia over 48 to 72 h [10]. As a consequence of these practical advantages, the ultrasound-guided superficial parasternal intercostal plane block (hereafter referred to as USG-PIFB) has gained rapid adoption as an adjunct to multimodal analgesia in cardiac anesthesia practice worldwide [1].

The evidence supporting USG-PIFB in cardiac surgery has grown considerably, with more than twenty randomized controlled trials published to date. Several trials have demonstrated reductions in postoperative pain scores and opioid requirements following bilateral USG-PIFB [3,10], while others—particularly those employing rigorous sham-controlled, blinded designs—have reported more modest or non-significant benefits [11]. This inconsistency across individual trials may reflect differences in study design (open-label versus blinded, sham versus no-block controls), block technique (single-shot versus catheter, single-level versus multi-level injection), local anesthetic pharmacology (drug, dose, concentration, and adjuvant use), and block timing relative to surgical incision. Concurrently, several alternative regional techniques targeting the parasternal territory have emerged as potential competitors, including the deep parasternal intercostal plane block (D-PIP, performed between the internal intercostal and transversus thoracis muscles), the transversus thoracic muscle plane block (TTMPB), and the erector spinae plane block (ESPB) [4,5]. How the superficial approach compares with these alternatives—and whether a meaningful dose–response relationship exists for local anesthetic dosing—remain open questions.

Two recent systematic reviews have attempted to synthesize the available evidence. Wardhana et al. [12] performed a focused meta-analysis restricted to opioid consumption and extubation time, without evaluating pain scores, recovery outcomes, or comparisons with alternative regional techniques. More comprehensively, Dost et al. [13] pooled randomized trials encompassing both ultrasound-guided fascial plane blocks and surgeon-performed parasternal wound infiltration. Their analysis reported a statistically significant but clinically modest reduction in 24-h opioid consumption (mean difference, −8.53 mg intravenous morphine milligram equivalents; 95% CI, −12.16 to −4.91), which fell below the threshold for minimal clinical importance and lost significance after correction for publication bias. Critically, the pooling of sonographically guided fascial plane blocks with surgeon-performed wound infiltration—techniques that differ in anatomical target, precision of drug delivery, and reproducibility—introduces substantial conceptual and statistical heterogeneity (I^2^ = 98.1%) that may obscure the true effect of the contemporary ultrasound-guided technique. Neither prior synthesis examined dose–response relationships, compared the superficial block with alternative parasternal or chest-wall techniques, or evaluated the relative merits of single-shot versus continuous catheter delivery.

We therefore conducted this systematic review and meta-analysis with a deliberately restricted scope: only randomized controlled trials employing ultrasound-guided superficial parasternal intercostal plane blocks were eligible, excluding surgeon-performed infiltration and landmark-based techniques. This restriction was designed to reduce technique-related heterogeneity and to generate estimates that are directly applicable to current anesthesiology practice. Our primary objective was to determine whether USG-PIFB reduces 24-h postoperative opioid consumption compared with sham, placebo, or no-block controls in adult cardiac surgery patients undergoing median sternotomy. Secondary objectives were to evaluate the effects of USG-PIFB on postoperative pain intensity at rest and during movement, extubation time, intensive care unit and hospital lengths of stay, postoperative nausea and vomiting, and rescue analgesic use. In a secondary analysis, we also aimed to compare USG-PIFB directly with alternative regional techniques (ESPB, D-PIP/TTMPB) where head-to-head trial data were available. Finally, we conducted dose–response meta-regression, subgroup analyses stratified by delivery method (single-shot versus catheter), comparator type (sham versus no-block), and block timing, and assessed the certainty of evidence using the Grading of Recommendations Assessment, Development and Evaluation (GRADE) framework.

## 2. Materials and Methods

### 2.1. Protocol and Registration

This systematic review and meta-analysis was conducted in accordance with the Preferred Reporting Items for Systematic Reviews and Meta-Analyses (PRISMA) 2020 statement [14] and was registered in the International Prospective Register of Systematic Reviews (PROSPERO; registration number: CRD420251114732) on 5 August 2025, before the literature search was performed. No separate full protocol was written. The registration was amended retrospectively (version 2.0, 9 March 2026), after the analyses had been completed, to align the record with the conduct of the review. Elements of the review that differ from the original registration are listed with their timing and rationale in Supplementary Material S1 (Table S1). In particular, the designation of 24-h opioid consumption as the sole primary outcome, the minimal clinically important difference thresholds, the trial sequential analysis, the trim-and-fill analysis and the dose–response meta-regression were not specified in the original registration and are reported as post hoc.

### 2.2. Eligibility Criteria

Studies were selected according to predefined Population, Intervention, Comparator, Outcomes, and Study design (PICOS) criteria.

#### 2.2.1. Population

Adult patients (aged ≥ 18 years) undergoing cardiac surgery via median sternotomy under general anesthesia. Studies exclusively enrolling pediatric populations or those performed through non-sternotomy approaches (minithoracotomy, robotic-assisted, or catheter-based procedures) were excluded.

#### 2.2.2. Intervention

Ultrasound-guided superficial pectointercostal plane (S-PIP) block, defined as deposition of local anesthetic in the fascial plane superficial to the intercostal muscles and deep to the pectoralis major muscle, performed under real-time sonographic visualization. This technique is variably described in the literature as the pectointercostal fascial block (PIFB), parasternal intercostal block (PIB), or superficial parasternal intercostal plane block. Studies employing surgeon-performed wound infiltration, landmark-based injection, or deep parasternal (transversus thoracic muscle plane) approaches without a concurrent superficial block arm were excluded. Both single-shot injection and continuous catheter-based delivery were eligible.

#### 2.2.3. Comparator

For the primary pairwise meta-analysis (Comparison 1), eligible comparators included: (a) sham or placebo injection (e.g., subcutaneous or fascial plane saline injection, dry needling, or saline delivered through identical catheters), (b) no block (standard systemic analgesia alone), or (c) active non-regional comparators, that is, an analgesic intervention delivered to the control arm but not to the block arm (e.g., drain-site local anesthetic infiltration or intravenous opioid infusion given to the control arm only). Co-interventions delivered identically to both arms, such as drain-site infiltration or multimodal systemic analgesia, did not change this classification, and trials in which both arms received a parasternal block, differing only in the continuation, drug or dose of the block, did not meet the comparator criterion. The classification of every trial contributing to the primary outcome is shown in Supplementary Material S2 (Table S2a,b). For the secondary comparative analysis (Comparison 2), studies directly comparing USG-guided S-PIP with an alternative regional technique—specifically, the erector spinae plane block (ESPB) or the deep parasternal intercostal plane block (D-PIP)/transversus thoracic muscle plane block (TTMPB)—were included regardless of whether a no-block control arm was present.

#### 2.2.4. Outcomes

The primary outcome was cumulative 24-h postoperative opioid consumption, expressed in intravenous morphine milligram equivalents (MME). Secondary outcomes included postoperative pain scores at rest and during movement (assessed at extubation, 6, 12, and 24 h on a 0–10 numeric rating scale), incidence of rescue analgesic use, time to first rescue analgesia, time to tracheal extubation, intensive care unit (ICU) and hospital lengths of stay, incidence of postoperative nausea and vomiting (PONV), and block-related complications (pneumothorax, local anesthetic systemic toxicity, hematoma). Complications were extracted separately for each arm and were considered block-attributable only when so judged by the trial investigators.

#### 2.2.5. Study Design

Only randomized controlled trials (RCTs), including parallel-group and multi-arm designs, were eligible. Non-randomized studies, quasi-experimental designs, case series, and conference abstracts without extractable quantitative data were excluded.

### 2.3. Information Sources and Search Strategy

A comprehensive systematic search was performed across six electronic databases: MEDLINE (Ovid), Embase (Ovid), the Cochrane Central Register of Controlled Trials (CENTRAL, via the Cochrane Library), CINAHL (EBSCOhost), Web of Science Core Collection, and Scopus (Elsevier). All databases were searched from inception through 31 December 2025, without language or publication-date restrictions. The search strategy was developed by the study team with input from a medical librarian and combined Medical Subject Headings (MeSH) and Emtree terms with free-text keywords across two concept domains: (a) pectointercostal or parasternal fascial plane block and its nomenclature variants (including S-PIP, SPIPB, PIFB, and parasternal intercostal block) and (b) cardiac surgery performed via sternotomy. Boolean operators and proximity operators (ADJ, NEAR/3, W/3) were adapted to each database interface. The full electronic search strategy for each database is provided in Supplementary Material S3.

In addition to the electronic database search, the following supplementary sources were screened: (a) ClinicalTrials.gov, the WHO International Clinical Trials Registry Platform (ICTRP), and the ISRCTN registry for ongoing or recently completed trials; (b) the reference lists of all included studies and relevant narrative reviews; and (c) forward citation tracking of key articles using Google Scholar. The gray literature search also included screening of preprint servers (medRxiv, Research Square) and conference proceedings of the European Society of Anaesthesiology and Intensive Care (ESAIC), the American Society of Anesthesiologists (ASA), and the Society of Cardiovascular Anesthesiologists (SCA).

The search was updated on 17 September 2026 using openly accessible sources: MEDLINE via PubMed and Europe PMC (including preprints) for records added from 1 August 2025 onwards, overlapping the period of the original search to allow for delayed indexing; Crossref metadata for journal articles published in the same period; and ClinicalTrials.gov without date restriction. The MEDLINE strategy was translated to PubMed syntax, and equivalent free-text strategies were used for Europe PMC and Crossref (Supplementary Material S3). Embase, CENTRAL, CINAHL, Web of Science and Scopus were not searched again. To verify completeness, the reference lists of newly included trials and of systematic reviews published between 2024 and 2026 were screened, the records retrieved by the original database search were re-screened with a structured keyword filter, MEDLINE was searched for randomized trials without date restriction, and Crossref was searched without date restriction for trials published in regional journals not indexed in the bibliographic databases.

### 2.4. Study Selection

**Figure 1:**
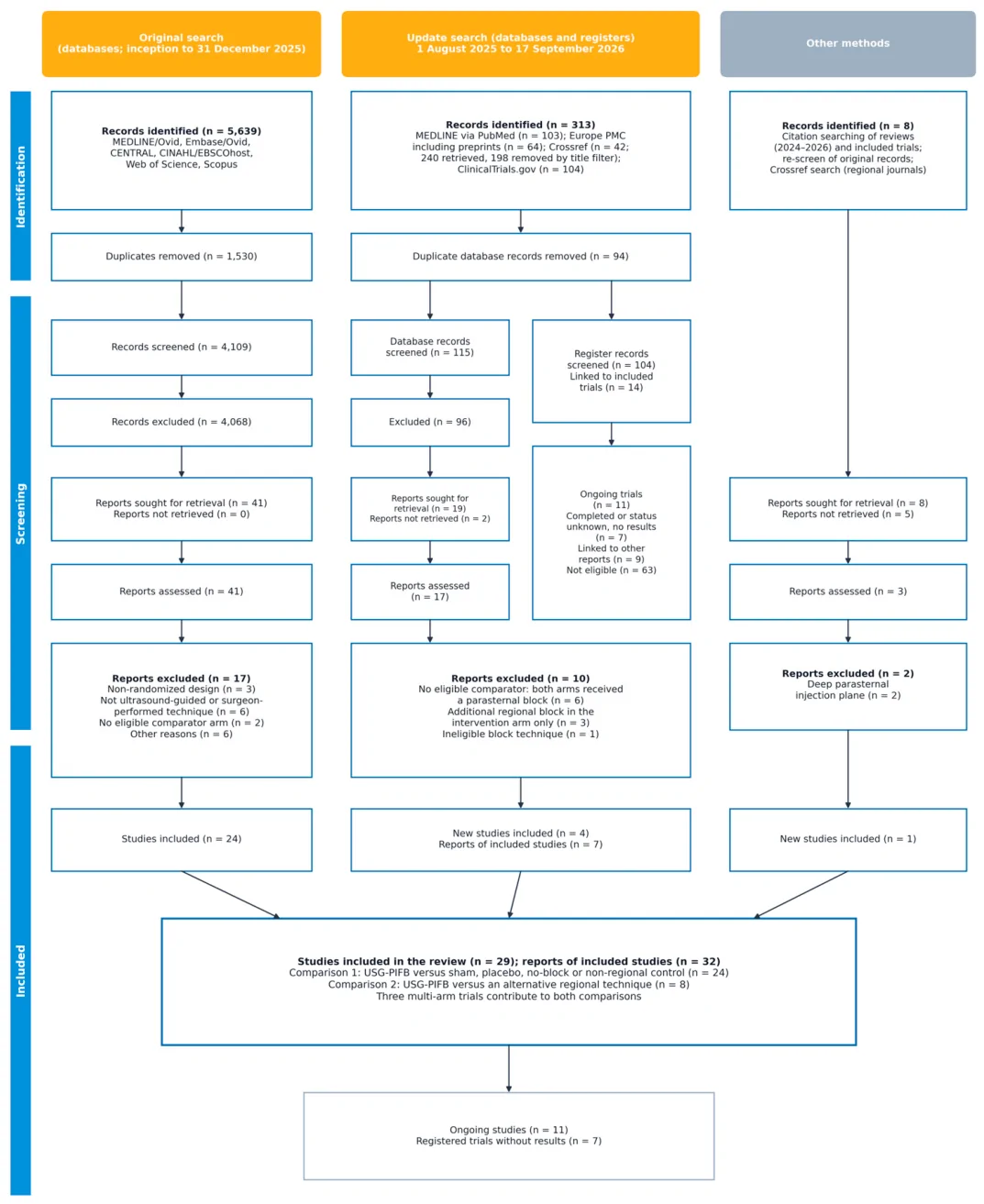
PRISMA 2020 flow diagram of the original search, the update search of databases and registers, and the other methods used during the update.

Records retrieved from all sources were imported into a reference management database and deduplicated using both automated algorithms and manual verification. Two reviewers independently screened titles and abstracts against the predefined eligibility criteria. Records judged as potentially relevant by either reviewer advanced to full-text assessment. Disagreements at the full-text stage were resolved by consensus or, when necessary, by adjudication with a third reviewer. Records from the update search were screened and assessed in the same way. Reasons for exclusion at the full-text stage were recorded and are reported in the PRISMA flow diagram (Figure 1) and in Supplementary Material S3. For multi-arm trials that included both a superficial block arm and a deep parasternal block arm (e.g., S-PIP vs. D-PIP vs. control), the S-PIP versus control comparison was included in Comparison 1, and the S-PIP versus D-PIP arm was analyzed separately in Comparison 2 to avoid double-counting of the shared control group.

### 2.5. Data Extraction

Data were extracted independently by two reviewers using a standardized, piloted data extraction form with 13 structured worksheets covering study characteristics, patient demographics, intervention details, anesthesia protocols, opioid consumption, pain scores, secondary outcomes, risk of bias assessments, Grading of Recommendations Assessment, Development and Evaluation (GRADE) domains, meta-regression covariates, and a conversion log. Discrepancies between reviewers were reconciled by discussion. Data from trials identified in the update search were extracted with the same form. Corresponding authors were contacted when published data were ambiguous or incomplete, and data reported only in the abstract of a companion report of an included trial were used when accompanied by measures of dispersion.

All opioid consumption data were converted to intravenous morphine milligram equivalents using standardized equianalgesic conversion factors derived from the European Society of Anaesthesiology and Intensive Care (ESAIC) and the American Pain Society guidelines (Supplementary Material S4); oral morphine equivalents were converted with a parenteral-to-oral ratio of 1:3, and oral oxycodone with a factor of 0.5, with the alternative intravenous factor examined in a sensitivity analysis. Continuous outcomes reported as median with interquartile range (IQR) were converted to mean and standard deviation (SD) using the method of Wan et al. [15], or the method of Luo et al. [16] for median with range; the same rule was applied to every outcome and every trial, except when the first and third quartiles were identical. Means reported with 95% confidence intervals were converted to SDs using the t distribution. Because these conversions assume approximately symmetric distributions, each continuous secondary outcome was also analyzed using only trials that reported means and SDs. Pain scores reported on a 0–100 visual analogue scale (VAS) were rescaled to a 0–10 numeric rating scale (NRS) by dividing by 10. When two eligible USG-PIFB arms shared a control arm, the arms were combined [17]; for the 2 × 2 factorial trial, USG-PIFB was compared with its placebo across both levels of the second factor, as reported by the investigators. When 24-h cumulative opioid consumption was not reported, the cumulative window closest to 24 h was used (Supplementary Material S2, Table S2b), and opioid consumption reported only over longer windows (72 h or 7 days) was analyzed as a standardized mean difference in a sensitivity analysis. Every data conversion was logged in a transparent conversion record to ensure full reproducibility.

### 2.6. Risk of Bias Assessment

Risk of bias was assessed for every included trial using the Cochrane Risk of Bias 2 (RoB 2) tool [18] across five domains: (D1) randomization process, (D2) deviations from intended interventions (effect of assignment), (D3) missing outcome data, (D4) measurement of the outcome, and (D5) selection of the reported result. For every domain of every trial, a structured support judgment documenting the relevant trial evidence was first recorded. Two assessors then rated all domains independently from these support judgments, the second assessor being blinded to the first assessor’s domain verdicts at the time of rating. Each domain was classified as low risk, some concerns, or high risk. The overall judgment followed the RoB 2 decision rules: low risk when all five domains were at low risk; high risk when at least one domain was at high risk, or when some concerns in multiple domains substantially lowered confidence in the result; and some concerns otherwise. The multiple-domain criterion was applied as some concerns in three or more domains; trials with some concerns in exactly two domains were judged individually, and the reasons for each of these judgments are documented in Supplementary Material S5. Inter-rater agreement before consensus was quantified using Cohen’s κ with a bootstrap 95% confidence interval (5000 resamples). Disagreements were resolved by discussion between the two assessors, with a third author adjudicating where consensus was not reached. Domain-level judgments, support statements and the full consensus log for all trials are provided in Supplementary Material S5.

### 2.7. Statistical Analysis

#### 2.7.1. Effect Measures and Pooling

Continuous outcomes (opioid consumption, pain scores, extubation time, length of stay) were synthesized as mean differences (MD) with 95% confidence intervals (CI). Dichotomous outcomes (rescue analgesic use, PONV) were pooled as odds ratios (OR) with 95% CI. All analyses—including every subgroup and sensitivity analysis—employed a random-effects model with restricted maximum likelihood (REML) estimation of between-study variance (τ^2^) and the Knapp–Hartung adjustment of the pooled confidence interval, which provides more conservative inference than the DerSimonian–Laird approach when the number of studies is modest [19]. The ad hoc modification of the Knapp–Hartung method was used, in which the variance scaling factor is not allowed to fall below one, so that intervals are never narrower than those of the conventional random-effects model [20]. The DerSimonian–Laird and Paule–Mandel estimators were used only within the sensitivity analyses described in Section 2.7.3. The 95% prediction interval (PI) was calculated for outcomes with at least three trials to convey the expected range of effects in a new clinical setting [21]. Subgroups containing very few trials necessarily yield wide confidence intervals, which are reported as estimated. For Comparison 2, random-effects pooling was performed only when outcome units and reporting formats permitted harmonization to MME without unverifiable assumptions; otherwise results were summarized narratively. Block-related complications were summarized by arm without pooling, because events were rare.

#### 2.7.2. Assessment of Heterogeneity

Between-study heterogeneity was quantified using the I^2^ statistic, interpreted according to conventional thresholds: low (<25%), moderate (25–75%), and substantial (>75%). Cochran’s Q test was used to assess statistical significance of heterogeneity at the α = 0.10 level. The τ^2^ estimate was reported alongside I^2^ to provide an absolute measure of heterogeneity on the outcome scale.

#### 2.7.3. Subgroup and Sensitivity Analyses

Three subgroup analyses were conducted for the primary outcome: (a) delivery method (single-shot injection vs. continuous catheter), (b) comparator type (sham/placebo vs. no-block control), and (c) block timing relative to surgery (preoperative vs. postoperative). These subgroups were specified in the protocol amendment; the original registration listed block technique variant, use of adjuvants and type of surgery (Supplementary Material S1, Table S1). Subgroup differences were evaluated using a mixed-effects test for interaction (Q-between) when each subgroup contained at least two trials. All three comparisons are between-study rather than within-trial contrasts; they are therefore interpreted throughout as exploratory and hypothesis-generating. A nominal interaction *p*-value below 0.05 would not, on its own, have been treated as establishing a genuine subgroup effect, and the presence of statistical significance in one subgroup but not another was not interpreted as evidence of a difference between subgroups.

Sensitivity analyses of the primary outcome comprised: (a) sequential exclusion of each study (leave-one-out); (b) exclusion of studies judged at high risk of bias, and restriction to studies judged at low risk of bias; (c) restriction to studies using sham or placebo comparators; (d) re-analysis using the DerSimonian–Laird and Paule–Mandel estimators; and (e) exclusion of studies whose primary-outcome data required distributional imputation (median-to-mean or confidence-interval-to-standard-deviation conversion), with a stricter variant additionally excluding any study that required equianalgesic unit conversion. Of these, exclusion of studies at high risk of bias and the use of an alternative estimator were specified in the original registration. The following analyses were added post hoc and are labelled as such: (f) restriction to trials reporting cumulative consumption over 0–24 h; (g) conversion of oxycodone as an intravenous rather than oral opioid in the only trial reporting oxycodone consumption, and exclusion of that trial; (h) a standardized mean difference analysis adding trials that reported cumulative opioid consumption only over longer windows; (i) exclusion of statistical outliers, defined as trials whose 95% confidence interval lay entirely outside the pooled confidence interval; and, for continuous secondary outcomes, (j) restriction to trials reporting means and SDs. All analyses are reported in Section 3.11.

#### 2.7.4. Meta-Regression

Univariate random-effects meta-regression was performed using REML estimation with the Knapp–Hartung modification to explore potential sources of heterogeneity in the primary outcome. Nine study-level covariates were examined: total local anesthetic dose (mg), total injectate volume (mL), local anesthetic concentration (%), use of an α_2_-agonist adjuvant, use of dexamethasone adjuvant, preoperative versus postoperative block timing, delivery mode (single-shot versus continuous catheter), sham versus no-block comparator, and use of patient-controlled analgesia; binary covariates with fewer than two trials in either category were not analyzed. Delivery mode was entered as one binary covariate because single-shot and catheter delivery are complementary categories of a single variable. Each covariate was tested individually and no multivariable model was fitted. Only 13 trials contributed to the primary outcome, so the ratio of trials to candidate covariates is very low and the analysis is materially vulnerable to overfitting, unstable coefficients and spurious associations. All meta-regression results are accordingly presented as exploratory and hypothesis-generating, and no covariate is claimed to be an effect modifier. R^2^ is reported as a descriptive quantity only. A dose–response meta-regression was additionally performed for total local anesthetic dose (concentration × volume) and, because the included agents are not equipotent on a milligram-for-milligram basis, was repeated within agent-specific strata.

#### 2.7.5. Publication Bias

Small-study effects were evaluated by inspection of contour-enhanced funnel plots and by Egger’s regression test [22] for outcomes pooling 10 or more studies. Duval and Tweedie’s trim-and-fill method [23] was applied post hoc as a sensitivity analysis, using the L0 and R0 estimators and the same random-effects model as the primary analysis; because the number of imputed studies depends on the model used, a fixed-effect implementation was also reported. Funnel-plot asymmetry has several possible causes besides publication bias, including genuine between-study heterogeneity, systematic methodological differences between small and large trials, and chance; trim-and-fill is also known to perform poorly when heterogeneity is extreme. Trim-and-fill results are therefore reported as sensitivity analyses for small-study effects and are not presented as unbiased estimates of the treatment effect. Trial registry records were screened to identify completed trials without available results and ongoing trials (Supplementary Material S3).

#### 2.7.6. Trial Sequential Analysis

An exploratory, post hoc trial sequential analysis (TSA) was performed for the primary outcome [24]. Because the analysis was not part of the original registration and no anticipated intervention effect had been specified in advance, the required information size (RIS) was calculated for a range of anticipated mean differences (5, 7.5, 10 and 15 mg intravenous morphine equivalents), with a two-sided type I error rate of 5%, 90% power, and the variance taken from the pooled control-group standard deviation. The RIS was adjusted for heterogeneity using the diversity D^2^ = (V_R − V_F)/V_R, where V_R and V_F are the variances of the random-effects and fixed-effect pooled estimates [25]. Lan–DeMets O’Brien–Fleming α-spending monitoring boundaries were calculated for the cumulative z-curve, with trials ordered by year of publication. Because the RIS becomes highly sensitive to its assumptions when diversity approaches 100%, the TSA was given less interpretive weight than the random-effects estimate, the prediction interval and the GRADE assessment, and no conclusion about the presence or absence of an effect was based on it.

#### 2.7.7. Certainty of Evidence Assessment

The certainty of evidence for each outcome was assessed using the GRADE approach [26], starting at high certainty for randomized evidence. Because minimal clinically important difference thresholds were not part of the original registration (Section 2.7.8), certainty was rated in a non-null effect, with the null as the threshold [27]. The following explicit criteria were applied uniformly to all outcomes. Risk of bias was rated serious when less than 50% of the random-effects analysis weight came from trials at overall low risk of bias, and very serious when at least 50% came from trials at high risk. Inconsistency was rated serious when I^2^ exceeded 50%, and very serious when I^2^ exceeded 75% with trial estimates on both sides of the null. Indirectness was judged against the population, intervention, comparator and outcome of the review question, including the observation windows of the outcome data. Imprecision was rated serious when the 95% CI included no effect or when fewer than 400 participants (or 300 events for dichotomous outcomes) contributed, and very serious when both applied or, for dichotomous outcomes, when the 95% CI included both a 25% relative reduction and a 25% relative increase [28]. Publication bias was rated as suspected when funnel-plot asymmetry with larger benefits in smaller trials was supported by Egger’s test (*p* < 0.10) or by a change in the statistical conclusion under trim-and-fill; for outcomes with fewer than 10 trials, small-study effects could not be assessed statistically and certainty was not downgraded for this domain. The judgment for every domain of every outcome, including the reasons for not downgrading, is reported in the GRADE evidence profile (Supplementary Material S6, Table S6a).

#### 2.7.8. Clinical Significance Thresholds

Minimal clinically important differences (MCID) of 10 mg intravenous morphine equivalents over 24 h for opioid consumption and of 1.0 point on the 0–10 NRS for pain were not specified in the original registration; they were added in the retrospective amendment (Supplementary Material S1, Table S1) and are therefore post hoc. They were used only as descriptive benchmarks when interpreting the size of pooled estimates and did not inform the GRADE ratings, the parameters of the trial sequential analysis or any inferential conclusion. The 10-mg threshold is based on consensus recommendations derived in total hip and knee arthroplasty populations [29], and no MCID for 24-h opioid consumption has been established for adult cardiac surgery. Patients after median sternotomy differ from arthroplasty patients in baseline opioid requirement, in the respiratory consequences of inadequate analgesia, and in the duration of mechanical ventilation and intensive care, so the amount of opioid sparing that is clinically meaningful may plausibly differ between these populations.

#### 2.7.9. Software

All statistical analyses were performed in Python (version 3.9) using a custom meta-analytic engine built on NumPy (v1.26) and SciPy (v1.13) for numerical computation and distributional inference, pandas (v1.5) for data handling, statsmodels (v0.14) and scikit-learn (v1.6) for auxiliary statistics, and Matplotlib (v3.9) for visualization. Between-study variance was estimated by REML throughout, with the ad hoc Knapp–Hartung variance correction applied to all pooled confidence intervals; the DerSimonian–Laird and Paule–Mandel estimators were used only in sensitivity analyses. Figures were generated at 300 dpi. Trial sequential analysis boundaries were computed by recursive numerical integration of the Lan–DeMets O’Brien–Fleming α-spending function. Statistical significance was defined as a two-sided *p* < 0.05 unless otherwise specified. To permit independent verification, the complete analysis code, the study-level analytical dataset, the opioid conversion table, the data-conversion log, the domain-level RoB 2 judgments with support statements, and the complete GRADE evidence profile are provided as Supplementary Materials S4–S6.

## 3. Results

### 3.1. Study Selection and Retrieval

The original database search identified 5639 records from six electronic databases (MEDLINE/Ovid, Embase/Ovid, CENTRAL via Cochrane Library, CINAHL/EBSCOhost, Web of Science, Scopus; search date: 31 December 2025). After removal of 1530 duplicates, 4109 records were screened by title and abstract, and 41 full-text reports were assessed for eligibility, of which 17 were excluded (non-randomized design, *n* = 3; non-ultrasound-guided or surgeon-performed technique, *n* = 6; no eligible comparator arm, *n* = 2; other reasons, *n* = 6). One of these exclusions concerned a trial in which both arms received the same initial parasternal block and only subsequent catheter boluses were placebo-controlled, so that continuous and single-shot delivery rather than USG-PIFB and a control were compared [30]. The update search identified 209 database records and 104 registry records. After removal of 94 duplicates, 115 database records were screened and 17 full-text reports were assessed, of which 10 were excluded (no eligible comparator because both arms received a parasternal block, *n* = 6; an additional regional block in the intervention arm only, *n* = 3; ineligible block technique, *n* = 1), and four new trials were included [31,32,33,34]; the three remaining reports were companion reports of included trials, one of which provided 24-h opioid consumption data for a trial identified by the original search [35]. Citation searching, re-screening of the original search records and the supplementary Crossref search identified one further eligible trial [36]; two further reports were excluded because the injection was made in the deep parasternal plane. In total, 29 unique randomized controlled trials were included in the systematic review [3,10,11,31,32,33,34,36,37,38,39,40,41,42,43,44,45,46,47,48,49,50,51,52,53,54,55,56,57]. The registry search identified 11 ongoing trials and 7 completed or status-unknown trials without available results (Supplementary Material S3). Of the 29 trials, 24 contributed to Comparison 1 (USG-PIFB versus sham, placebo, no-block or active non-regional control) and eight to Comparison 2 (USG-PIFB versus an alternative regional technique). Five trials (Elbardan 2024 [55], Kaya 2022 [53], Keshwani 2023 [57], Mansour 2024 [54], Vanjare 2025 [36]) were dedicated head-to-head trials without a control arm and contributed to Comparison 2 only; three multi-arm trials (Demarquette 2025 [45], Toker 2025 [56], Yadav 2024 [43]) contributed a S-PIP-versus-control comparison to Comparison 1 and a S-PIP-versus-alternative-block comparison to Comparison 2. The number of unique trials (24 + 5 = 29) therefore differs from the number of trials contributing to the two comparisons (24 + 8 = 32). Multi-arm trials were never counted as independent studies within a single analysis, and their shared control arm was not double-counted. A study-level map showing which trial contributed to which comparison and outcome is provided in Supplementary Material S2 (Table S2a). The PRISMA 2020 flow diagram is presented in Figure 1.

### 3.2. Study Characteristics

Across the 29 trials, 2812 patients were randomized and 2664 contributed data to this review; the remaining 148 were randomized to trial arms that did not meet our eligibility criteria (for example, the bupivacaine-plus-clonidine arm of Singh 2024 [38] and the third arm of Eloraby 2024 [48]) or were excluded from the analyzed populations of the trials. Of the 2664 contributing participants, 2156 (1090 assigned to USG-PIFB and 1066 to control) were enrolled in the 24 Comparison 1 trials, 378 were enrolled in the five dedicated head-to-head trials, and a further 130 were enrolled in the alternative-block arms of the three multi-arm trials. Not every trial reported every outcome, so the number of participants contributing to each individual meta-analysis is smaller than these totals and is reported separately for each outcome below. The 24 Comparison 1 trials were conducted in 10 countries: China (*n* = 5), India (*n* = 5), Egypt (*n* = 4), Canada (*n* = 2), France (*n* = 2), the United States (*n* = 2), Finland (*n* = 1), Indonesia (*n* = 1), Italy (*n* = 1), and Türkiye (*n* = 1). All but one trial (Alli 2026 [31], four centres) were single-centre. Ropivacaine was used in 14 trials, plain bupivacaine in seven, bupivacaine mixtures (liposomal with plain bupivacaine; bupivacaine with lidocaine) in two, and levobupivacaine in one. Total injectate volumes ranged from 30 to 60 mL, and concentrations of plain local anesthetics from 0.125% to 0.75%. Perineural adjuvants were used in six trials: dexamethasone (*n* = 2), epinephrine (*n* = 2), fentanyl (*n* = 1), and a combination of dexamethasone, dexmedetomidine and epinephrine (*n* = 1). Eleven trials used a sham or placebo comparator, eleven compared the block with no block, and two used an active non-regional comparator. Three trials used catheter delivery with programmed intermittent bolus or continuous infusion over 48 to 72 h. The detailed study characteristics are presented in Table 1, and the comparator classification of the trials contributing to the primary outcome in Supplementary Material S2 (Table S2b).

### 3.3. Risk of Bias Assessment in Included Studies

**Figure 2:**
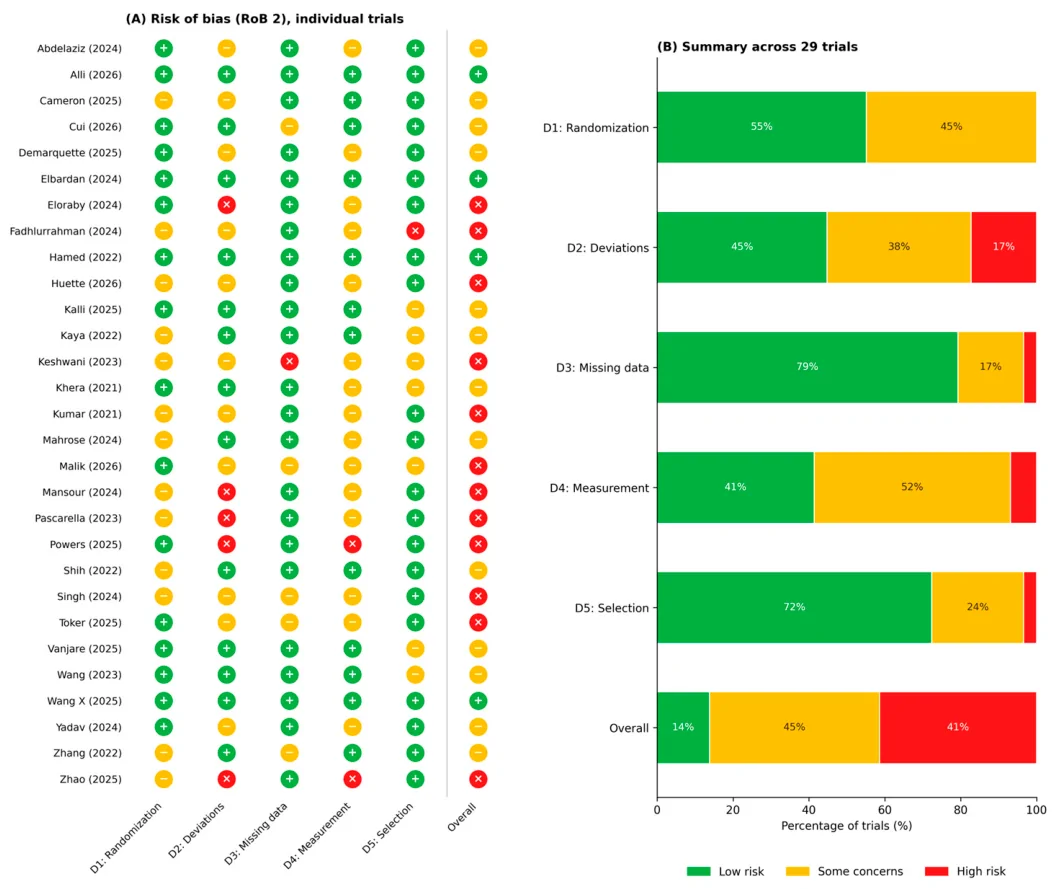
Risk of bias assessment using the Cochrane Risk of Bias 2 (RoB 2) tool. (**A**) Consensus domain-level and overall judgments for each included trial across five domains: randomization process (D1), deviations from intended interventions (D2), missing outcome data (D3), measurement of the outcome (D4), and selection of the reported result (D5). Green (+), low risk; yellow (−), some concerns; red (×), high risk. (**B**) Proportion of trials at each risk level by domain and overall. Trials shown in (**A**): Abdelaziz (2024) [39], Alli (2026) [31], Cameron (2025) [47], Cui (2026) [33], Demarquette (2025) [45], Elbardan (2024) [55], Eloraby (2024) [48], Fadhlurrahman (2024) [40], Hamed (2022) [41], Huette (2026) [34], Kalli (2025) [42], Kaya (2022) [53], Keshwani (2023) [57], Khera (2021) [11], Kumar (2021) [44], Mahrose (2024) [3], Malik (2026) [32], Mansour (2024) [54], Pascarella (2023) [37], Powers (2025) [49], Shih (2022) [50], Singh (2024) [38], Toker (2025) [56], Vanjare (2025) [36], Wang (2023) [51], Wang X (2025) [46], Yadav (2024) [43], Zhang (2022) [52], Zhao (2025) [10].

Risk of bias was assessed with the Cochrane Risk of Bias 2 (RoB 2) tool for all 29 included trials, comprising both comparisons (Figure 2). For the 24 trials identified by the original search, the two assessors rated all 120 domain judgments independently and agreed on 115 (95.8%), giving a Cohen’s κ of 0.92 (bootstrap 95% CI, 0.84 to 0.98); they agreed on the overall judgment for 23 of 24 trials (κ = 0.93). The five trials identified during the search update were assessed in the same way. Applying the RoB 2 decision rules, 4 of the 29 trials (14%) were at overall low risk of bias, 13 (45%) raised some concerns, and 12 (41%) were at high risk of bias. Five trials were rated at high risk because of some concerns in three or more domains without any domain at high risk (Huette 2026 [34], Kumar 2021 [44], Malik 2026 [32], Singh 2024 [38], Toker 2025 [56]), and eight trials with some concerns in exactly two domains were judged individually and remained at some concerns (Supplementary Material S5). At domain level, concerns were most frequent in deviations from intended interventions (D2: 13 low, 11 some concerns, 5 high) and measurement of the outcome (D4: 12 low, 15 some concerns, 2 high), reflecting the absence of a sham injection in many trials and the subjective nature of pain scoring, followed by the randomization process (D1: 16 low, 13 some concerns, 0 high), mostly because allocation concealment was not reported; missing outcome data (D3: 23 low, 5 some concerns, 1 high) and selection of the reported result (D5: 21 low, 7 some concerns, 1 high) were less often problematic. Other trials were rated at high risk because of asymmetric co-interventions between arms (Powers 2025 [49], Pascarella 2023 [37]), open-label designs without any sham procedure (Zhao 2025 [10], Eloraby 2024 [48], Mansour 2024 [54]), absence of trial registration combined with selective emphasis of results (Fadhlurrahman 2024 [40]), or attrition exceeding 20% (Keshwani 2023 [57]). Of the 13 trials contributing to the primary outcome, 2 were at low risk, 9 raised some concerns and 2 were at high risk of bias. Complete domain-level judgments, support statements and the consensus log are provided in Supplementary Material S5.

### 3.4. Primary Outcome: 24-H Opioid Consumption

#### 3.4.1. Main Analysis

**Figure 3:**
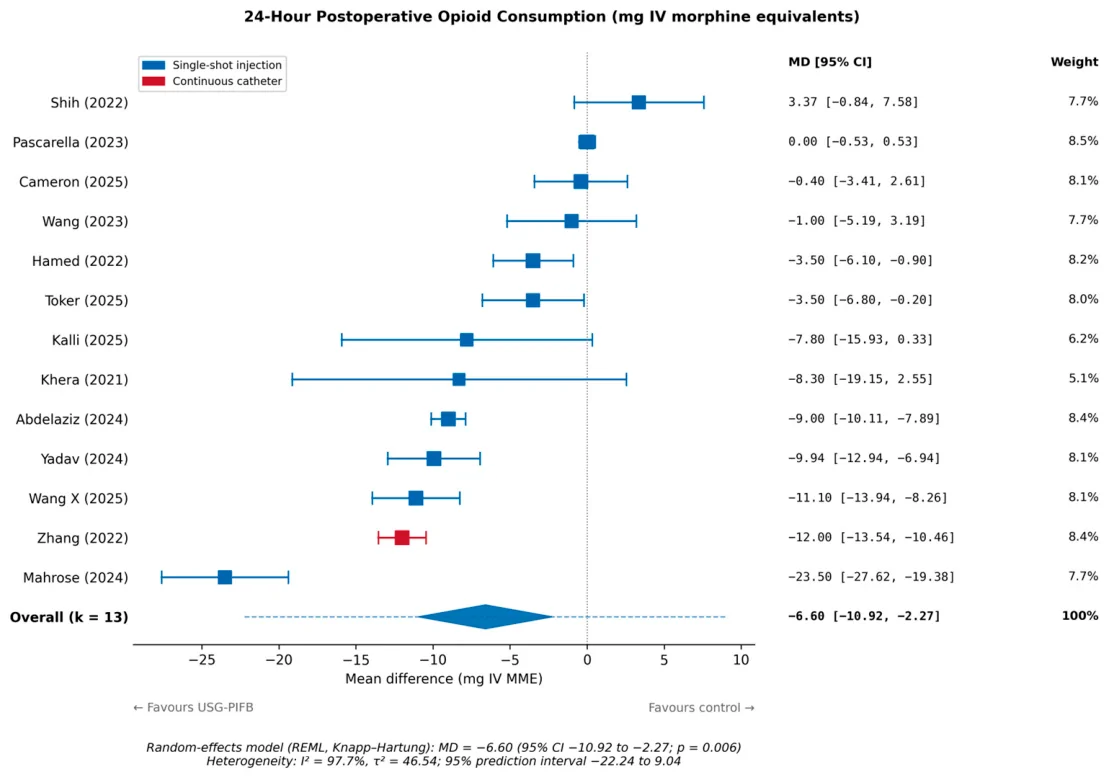
Forest plot of 24-h postoperative opioid consumption (mg intravenous morphine milligram equivalents) comparing ultrasound-guided pectointercostal fascial block (USG-PIFB) with control. Square size is proportional to study weight. Blue squares denote single-shot injection and the red square continuous catheter delivery. The diamond represents the pooled random-effects estimate (REML with Knapp–Hartung adjustment) and the dashed line the 95% prediction interval. Trials shown (top to bottom): Shih (2022) [50], Pascarella (2023) [37], Cameron (2025) [47], Wang (2023) [51], Hamed (2022) [41], Toker (2025) [56], Kalli (2025) [35,42], Khera (2021) [11], Abdelaziz (2024) [39], Yadav (2024) [43], Wang X (2025) [46], Zhang (2022) [52], Mahrose (2024) [3].

Thirteen studies comprising 902 patients (450 in the USG-PIFB group and 452 in the control group) reported 24-h postoperative opioid consumption in intravenous morphine milligram equivalents (MME). The pooled analysis showed a reduction in 24-h opioid consumption with USG-PIFB, with a mean difference of −6.60 mg (95% CI, −10.92 to −2.27; *p* = 0.006; Figure 3). Between-study heterogeneity was extreme (I^2^ = 97.7%; τ^2^ = 46.54). The 95% prediction interval ranged from −22.24 to 9.04 mg, indicating that in a new clinical setting the effect could plausibly range from a substantial reduction to no reduction or a small increase in opioid consumption. The pooled mean therefore describes an average across heterogeneous trials rather than an effect that can be expected in every setting. As a descriptive, post hoc benchmark (Section 2.7.8), the pooled reduction was smaller than 10 mg, and its confidence interval included reductions of less than 3 mg as well as reductions of about 11 mg.

#### 3.4.2. Publication Bias Assessment

**Figure 4:**
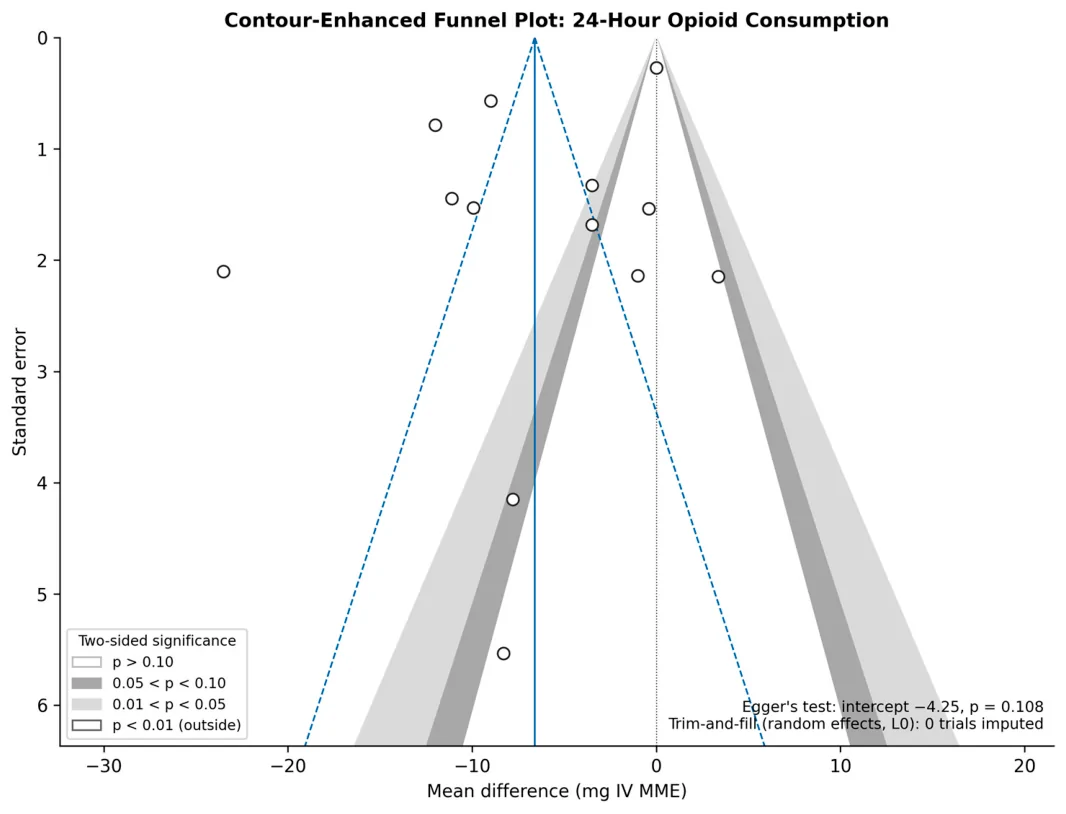
Contour-enhanced funnel plot for the primary outcome (24-h opioid consumption). Each point represents one study, plotted against its standard error. Shaded regions indicate two-sided significance levels around zero; dashed lines indicate pseudo 95% confidence limits around the pooled random-effects estimate (solid vertical line).

Visual inspection of the funnel plot suggested asymmetry, with smaller trials tending to report larger reductions (Figure 4). Egger’s test did not reach statistical significance (intercept −4.25; *p* = 0.11), and its power is limited with 13 studies. Trim-and-fill based on the random-effects model imputed no studies with either the L0 or the R0 estimator, leaving the estimate unchanged. A fixed-effect implementation imputed 6 studies and moved the estimate to −0.22 mg (95% CI, −0.59 to 0.16), or to −0.78 mg (95% CI, −6.10 to 4.53) when the augmented data were re-analyzed with the random-effects model. Neither result constitutes a correction for publication bias. With heterogeneity this extreme (I^2^ = 97.7%), funnel-plot asymmetry is at least as likely to reflect genuine between-study differences—in local anesthetic regimen, comparator design, background analgesia and institutional practice—as selective publication, and the fixed-effect implementation ignores this heterogeneity. The divergence between implementations nevertheless indicates that the primary estimate is sensitive to plausible small-study effects, and publication bias was rated as suspected in the GRADE assessment.

#### 3.4.3. Trial Sequential Analysis Findings

In the exploratory trial sequential analysis, the pooled control-group standard deviation was 11.2 mg and the diversity was D^2^ = 98.8%. For an anticipated difference of 10 mg, the unadjusted required information size was 53 participants and the heterogeneity-adjusted size 4419 participants, of which the accrued 902 participants represent 20%. The cumulative z-curve (final z = −3.32) crossed the conventional significance boundary but not the monitoring boundary (|z| = 4.36 at the final analysis). The monitoring boundaries were likewise not crossed for anticipated differences of 5 and 7.5 mg, whereas for 15 mg—more than twice the pooled estimate—the final boundary (|z| = 2.97) was crossed (Supplementary Material S6, Table S6b and Figure S1). Because these results depend on the assumed effect and on a diversity estimate close to 100%, the analysis does not establish whether or not USG-PIFB reduces opioid consumption across the range of settings represented here, and it was not used to support any conclusion.

### 3.5. Pain Scores

#### 3.5.1. Pain at Rest

**Figure 5:**
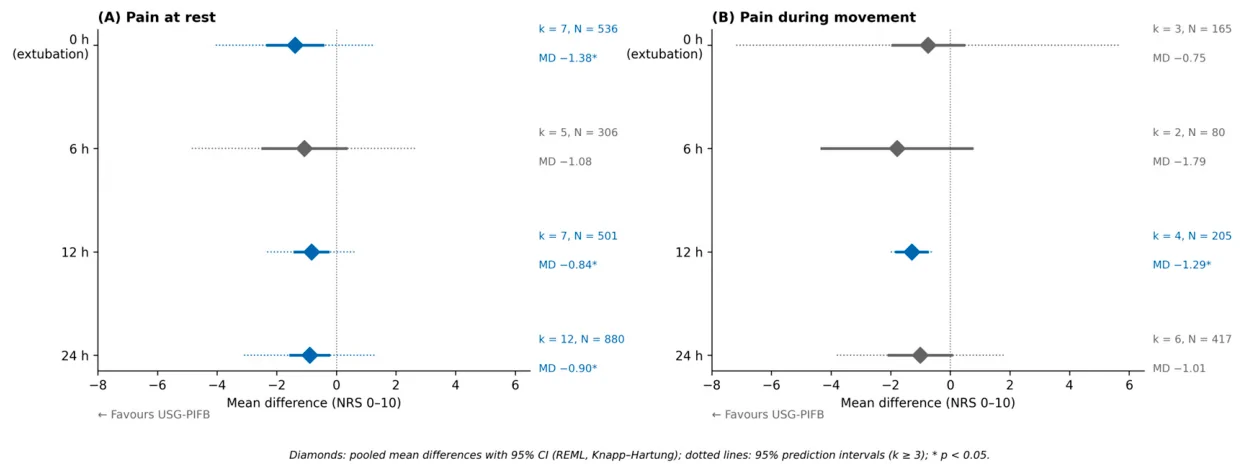
Summary of pooled pain score analyses at extubation and at 6, 12 and 24 h. (**A**) Pain at rest. (**B**) Pain during movement. Diamonds represent pooled mean differences with 95% confidence intervals (REML with Knapp–Hartung adjustment); dotted lines indicate 95% prediction intervals where available; blue markers and asterisks denote *p* < 0.05.

Postoperative pain at rest was assessed at four time points across 5 to 12 studies per time point (Table 2 and Figure 5). USG-PIFB was associated with lower pain scores at extubation (MD −1.38; 95% CI, −2.31 to −0.45; *p* = 0.01; k = 7), at 12 h (−0.84; 95% CI, −1.39 to −0.28; *p* = 0.01; k = 7) and at 24 h (−0.90; 95% CI, −1.54 to −0.26; *p* = 0.01; k = 12), but not at 6 h (−1.08; 95% CI, −2.48 to 0.32; *p* = 0.10; k = 5). Heterogeneity was substantial at every time point (I^2^ 79% to 97%), and all prediction intervals crossed the line of no effect. When the analysis was restricted to trials reporting means and standard deviations, the point estimates were similar, but statistical significance was retained only at 12 h (Table 2), indicating that the precision of these estimates partly depends on median-to-mean conversions.

#### 3.5.2. Pain During Movement

Pain during movement was evaluated in 2 to 6 studies per time point. It was lower at 12 h (MD −1.29; 95% CI, −1.81 to −0.77; *p* = 0.004; I^2^ = 0.0%; k = 4; 205 participants), but not significantly at extubation (−0.75; 95% CI, −1.94 to 0.45; k = 3), 6 h (−1.79; 95% CI, −4.31 to 0.73; k = 2) or 24 h (−1.01; 95% CI, −2.05 to 0.04; I^2^ = 92.4%; k = 6). The small number of trials and participants at the earlier time points limits these estimates. At rest and during movement, all pooled differences were smaller than 1.5 points on the 0–10 scale, and the pattern across time points did not show a consistent difference between resting and dynamic pain.

### 3.6. Secondary Outcomes

#### 3.6.1. Rescue Analgesics

Six studies (*n* = 596 patients) reported the incidence of rescue analgesic use. The pooled odds ratio favored USG-PIFB but did not reach statistical significance (OR 0.61; 95% CI, 0.32 to 1.15; *p* = 0.10; I^2^ = 31.3%). Time to first rescue analgesia was reported by four trials, in each of which it was longer after USG-PIFB, by approximately 6 to 13 h; because reporting formats differed, this outcome was summarized narratively.

#### 3.6.2. Extubation Time and Length of Stay

Sixteen studies (*n* = 1563 patients) reported time to tracheal extubation. The pooled analysis showed a reduction of −106.1 min (95% CI, −174.4 to −37.9; *p* = 0.005; I^2^ = 97.5%) with USG-PIFB. However, the extreme heterogeneity and wide 95% prediction interval (−374.3 to 162.0 min), which crossed the line of no effect, indicate that this finding should be regarded as exploratory; the analysis restricted to trials reporting means gave −140.9 min (k = 12). The direction and magnitude of the effect may vary substantially across clinical settings, depending on institutional extubation protocols, sedation practices, and surgeon preferences.

Fourteen studies (*n* = 1496 patients) contributed to the ICU length of stay analysis, which was shorter with USG-PIFB (MD −13.4 h; 95% CI, −21.3 to −5.4; *p* = 0.003; I^2^ = 92.3%; 95% PI −41.8 to 15.1 h); the direction was unchanged in the analysis restricted to trials reporting means (−17.7 h; k = 8). Hospital length of stay, reported in ten studies (*n* = 1116 patients), did not differ significantly between groups (MD −0.66 days; 95% CI, −1.51 to 0.19; *p* = 0.11; I^2^ = 92.2%), although the analysis restricted to trials reporting means suggested a reduction (−1.48 days; 95% CI, −2.73 to −0.24; k = 5).

#### 3.6.3. Postoperative Nausea and Vomiting

Seven studies (*n* = 720 patients) evaluated the incidence of PONV. The pooled odds ratio was not statistically significant (OR 0.62; 95% CI, 0.24 to 1.61; *p* = 0.27; I^2^ = 74.5%), and the estimate was too imprecise to exclude either a clinically relevant reduction or an increase in PONV with USG-PIFB.

#### 3.6.4. Block-Related Adverse Events

Block-related complications were reported, at least in part, by 19 of the 29 included trials; a general statement that no block-related complications had occurred was counted as zero events for each complication type. No episode of local anesthetic systemic toxicity was reported in any arm of the 16 trials reporting this outcome (0/606 USG-PIFB and 0/609 comparator patients); by the rule of three, this is compatible with a true incidence of up to approximately 0.5% after USG-PIFB. No pneumothorax was attributed to the superficial block by the trial investigators. Pneumothorax was reported as a postoperative complication in two trials, at similar frequencies in the block and control arms (Demarquette 2025 [45], 4/84 and 2/85; Cui 2026 [33], 4/126 and 6/134; overall 8/732 and 8/743 in the 17 trials reporting this outcome); in the three-arm trial by Demarquette et al. [45] it was more frequent after the deep parasternal block (10/85), and the adverse effects attributed to ropivacaine (2/84 after USG-PIFB and 2/85 after the deep block) comprised nausea and vomiting and one episode of arterial hypotension after the deep block. One block-site hematoma occurred in a patient who received USG-PIFB (Abdelaziz 2024 [39], 1/20), compared with three injection-site hematomas after sham injection in the same trial (3/20). Ten trials did not report block-related complications, and the largest trial (Alli 2026 [31]) reported only serious block-related complications, of which there were none. Because events were rare, formal meta-analysis was not performed. These data indicate that no safety signal emerged for USG-PIFB in the randomized literature, but they do not permit the conclusion that USG-PIFB is safer than comparator techniques: no trial was powered for a safety endpoint, complication reporting was incomplete, and no trial systematically screened for asymptomatic complications. A study-level table of all reported adverse events is provided in Supplementary Material S7 (Table S7).

### 3.7. Head-to-Head Comparison: S-PIP Versus Alternative Regional Techniques

#### 3.7.1. S-PIP Versus Erector Spinae Plane Block

Two studies (Elbardan 2024 [55], *n* = 100; Keshwani 2023 [57], *n* = 38) directly compared USG-PIFB with ESPB in cardiac surgery patients. Both studies reported non-significant differences in pain scores between the two techniques at most time points. Elbardan et al. [55] reported higher 24-h opioid consumption in the PIFB group compared with ESPB, with the ESPB group showing longer time to first analgesic request. Keshwani et al. [57] found no significant difference in 24-h fentanyl consumption (PIFB 377.5 ± 93.3 µg vs. ESPB 435.4 ± 100.9 µg; *p* = 0.11). Although both trials compared USG-PIFB directly with ESPB, quantitative pooling of 24-h opioid consumption was not performed. The trials reported opioid use using non-commensurate metrics and formats (absolute fentanyl dose reported as mean ± SD versus weight-based fentanyl dose reported as median [IQR]), and the ESPB trial reporting weight-based consumption did not provide the underlying body-weight data necessary to convert to a common unit without additional assumptions. Additionally, the direction of effect differed between studies. Accordingly, the available evidence is insufficient to determine whether USG-PIFB is as effective as ESPB. Failure to demonstrate a difference between two techniques is not evidence that they are equivalent.

#### 3.7.2. S-PIP Versus Deep Parasternal Intercostal Plane Block/Transversus Thoracic Muscle Plane Block

**Figure 6:**
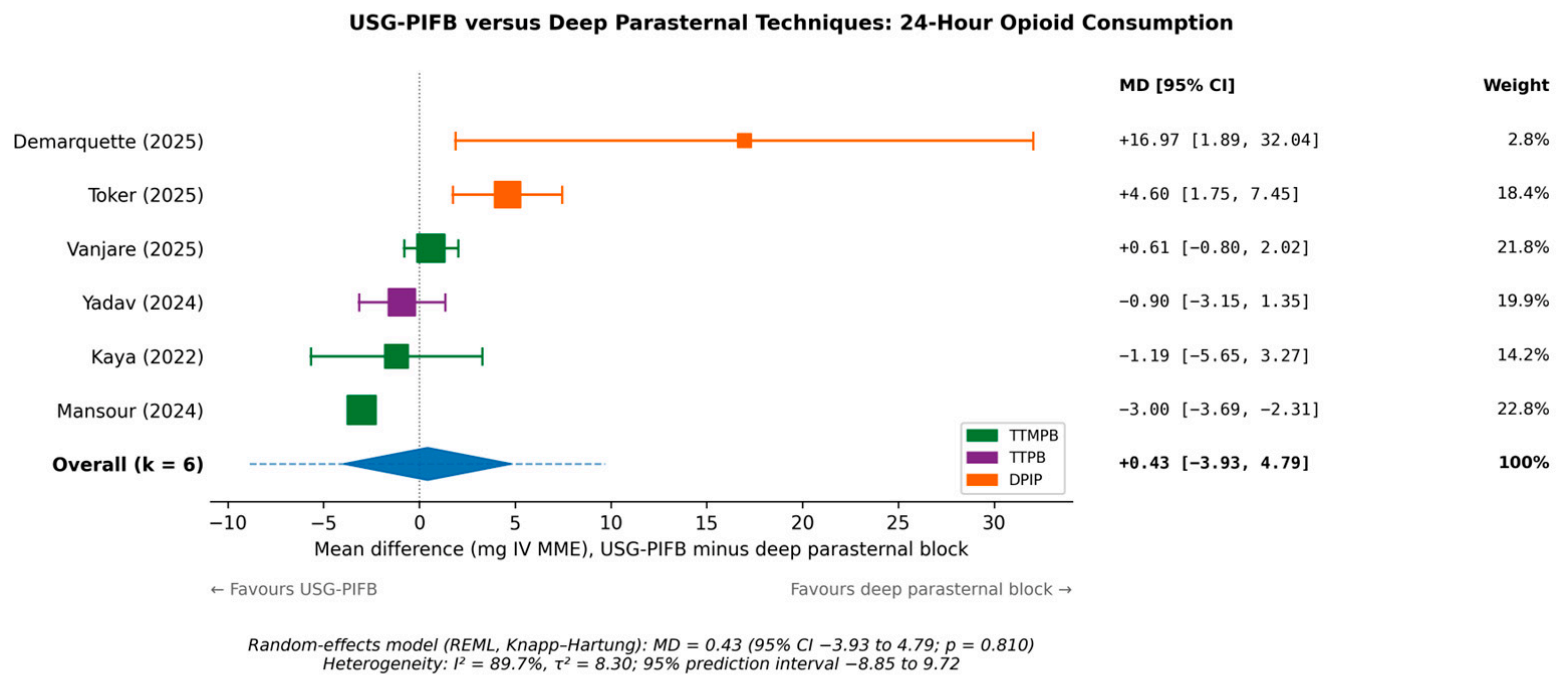
Forest plot of 24-h opioid consumption (mg IV morphine milligram equivalents) comparing USG-PIFB with deep parasternal techniques (TTMPB, TTPB, D-PIP). Positive values indicate higher consumption with USG-PIFB. Square size reflects study weight; horizontal lines indicate 95% CIs; the diamond indicates the pooled random-effects estimate (REML with Knapp–Hartung adjustment) and the dashed line the 95% prediction interval. Trials shown (top to bottom): Demarquette (2025) [45], Toker (2025) [56], Vanjare (2025) [36], Yadav (2024) [43], Kaya (2022) [53], Mansour (2024) [54].

Six studies compared USG-PIFB with D-PIP or TTMPB, including three dedicated head-to-head trials (Kaya 2022 [53], *n* = 42; Mansour 2024 [54], *n* = 80; Vanjare 2025 [36], *n* = 118) and three multi-arm studies with both superficial and deep parasternal block arms (Demarquette 2025 [45], Toker 2025 [56], Yadav 2024 [43]). Kaya et al. [53] reported no significant difference in 24-h morphine consumption between PIFB and TTMPB (13.89 ± 6.80 vs. 15.08 ± 7.42 mg; *p* = 0.608), Mansour et al. [54] found significantly lower rescue morphine use in the PIFB group compared with TTMPB (4.8 ± 1.0 vs. 7.8 ± 2.0 mg; *p* < 0.001), and Vanjare et al. [36] found similar 24-h tramadol consumption (194.8 ± 35.0 vs. 188.7 ± 42.6 mg; *p* = 0.672). Random-effects pooling of 24-h opioid consumption across the six studies (*n* = 496) showed no statistically significant difference between techniques (MD 0.43 mg MME; 95% CI, −3.93 to 4.79; *p* = 0.81; I^2^ = 89.7%; Figure 6), with effect directions split evenly between trials. In a sensitivity analysis excluding the single study requiring median-to-mean conversion, results remained null (MD −0.07 mg MME; 95% CI, −3.68 to 3.53; k = 5).

### 3.8. Dose–Response Analysis

**Figure 7:**
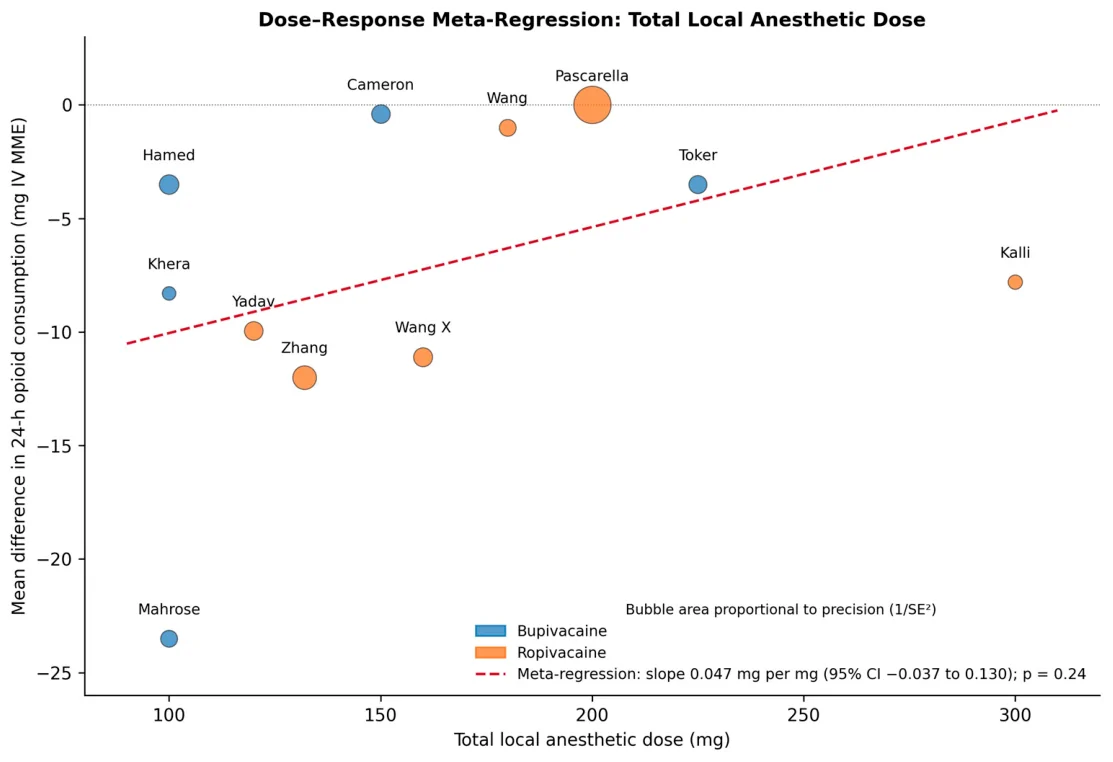
Dose–response bubble plot illustrating the relationship between total local anesthetic dose (mg) and the mean difference in 24-h opioid consumption. Bubble area is proportional to study precision (1/SE^2^); colors indicate the local anesthetic. The dashed red line represents the univariate meta-regression line (slope = 0.047; 95% CI −0.037 to 0.130; *p* = 0.24). The relationship is not statistically significant, and the positive slope is directionally opposite to a dose-dependent analgesic benefit. Agent-specific strata (bupivacaine, k = 5; ropivacaine, k = 6) are reported in Section 3.8. Trials shown: Cameron (2025) [47], Hamed (2022) [41], Kalli (2025) [35,42], Khera (2021) [11], Mahrose (2024) [3], Pascarella (2023) [37], Toker (2025) [56], Wang (2023) [51], Wang X (2025) [46], Yadav (2024) [43], Zhang (2022) [52].

A dose–response meta-regression was performed to explore the relationship between total local anesthetic dose (calculated as concentration × total volume, in milligrams) and the magnitude of opioid reduction at 24 h. Eleven of the 13 trials reported sufficient information to compute a total dose (range 100 to 300 mg); the remaining two used local anesthetic mixtures for which a single total dose is not meaningful. The regression slope was 0.047 mg MME per mg of local anesthetic (95% CI, −0.037 to 0.130; *p* = 0.24), a non-significant relationship whose point estimate is directionally opposite to a dose-dependent benefit: higher doses were associated with slightly less, not more, opioid sparing (Figure 7). The model accounted for a descriptive R^2^ of 9.2% of between-study variance, which we do not interpret as evidence that dose explains heterogeneity.

When volume and concentration were examined independently, neither was a statistically significant predictor of effect size (volume: slope = 0.31, *p* = 0.17; concentration: slope = 7.69, *p* = 0.63). Because the included trials used agents that are not equipotent on a milligram-for-milligram basis—bupivacaine, ropivacaine, levobupivacaine, liposomal bupivacaine and local anesthetic mixtures—pooling them on a total-milligram scale could in principle obscure or manufacture a dose–response relationship. We therefore repeated the analysis within agent-specific strata. The eleven trials with a computable total dose comprised 5 bupivacaine trials and 6 ropivacaine trials. Neither stratum showed a significant dose–response relationship (bupivacaine: slope = 0.08, 95% CI −0.21 to 0.36, *p* = 0.45; ropivacaine: slope = 0.04, 95% CI −0.08 to 0.16, *p* = 0.43), and in both strata the point estimate was positive, that is, directionally opposite to a dose-dependent benefit. Pooled effects were also similar for the two agents (ropivacaine: MD −6.96 mg, 95% CI −12.82 to −1.10, k = 6; bupivacaine: MD −7.74 mg, 95% CI −19.62 to 4.14, k = 5). The absence of a dose–response signal is therefore not an artefact of pooling non-equipotent agents. These analyses nonetheless rest on five and six trials, are ecological in nature, and should be regarded as exploratory; they cannot exclude a true dose–response relationship that a within-trial dose-finding design would detect.

### 3.9. Subgroup Analyses

#### 3.9.1. Delivery Method: Single-Shot Versus Continuous Catheter

SSubgroup analysis by delivery method is summarized in Table 3. Twelve trials used single-shot injection and yielded a pooled mean difference of −6.11 mg (95% CI, −10.74 to −1.48; I^2^ = 97.2%). Only one trial contributing to the primary outcome used continuous catheter delivery against a placebo infusion (Zhang 2022 [52]; MD −12.00 mg; 95% CI, −13.54 to −10.46), so a test for subgroup differences was not performed and no conclusion about the relative efficacy of catheter and single-shot delivery can be drawn from these data. The largest catheter trial (Alli 2026 [31]) reported opioid consumption only over 72 h and contributed to the standardized mean difference sensitivity analysis (Section 3.11). Any comparison of delivery methods across trials would in any case be a between-study rather than a randomized within-trial comparison.

#### 3.9.2. Comparator Type: Sham/Placebo Versus No-Block Control

Among trials using a sham or placebo comparator (k = 9) the pooled mean difference was −8.12 mg (95% CI, −14.19 to −2.06; I^2^ = 93.8%), while among trials using a no-block control (k = 4) it was −3.36 mg (95% CI, −10.67 to 3.94; I^2^ = 93.2%). The test for interaction was not statistically significant (*p* = 0.17). Because the interaction test is negative, the fact that the pooled estimate reaches statistical significance in one subgroup and not in the other cannot be interpreted as evidence that the effect genuinely differs between blinded and unblinded trials, and we make no such claim. Both subgroup estimates are compatible with a common underlying effect, and the difference between them is within the range expected from sampling variation given the small number of trials and the extreme heterogeneity present in both subgroups. None of the trials contributing to the primary outcome used an active non-regional comparator.

#### 3.9.3. Block Timing: Preoperative Versus Postoperative

Nine trials administered the block preoperatively and four postoperatively. The preoperative subgroup yielded a pooled mean difference of −7.89 mg (95% CI, −14.04 to −1.73; I^2^ = 98.5%) and the postoperative subgroup −2.69 mg (95% CI, −5.91 to 0.54; I^2^ = 22.0%), with no significant interaction (*p* = 0.07). Khera 2021 [11] performed the block postoperatively, within two hours of intensive care unit admission (Table 1), and is classified accordingly. As with the other subgroup analyses, this comparison is exploratory and was not powered to detect timing-related differences.

### 3.10. Meta-Regression Findings

Univariate meta-regression analyses were performed on the primary outcome to explore potential sources of heterogeneity (Table 4). Seven of the nine covariates could be examined; use of an α_2_-agonist adjuvant and delivery mode could not, because only one trial fell in one of the two categories. None of the seven covariates showed a statistically significant association with effect size. The largest descriptive R^2^ was observed for use of patient-controlled analgesia (slope = −7.83 mg, 95% CI −16.02 to 0.35; *p* = 0.059; R^2^ = 25.4%), where trials using patient-controlled delivery reported larger opioid-sparing effects. We do not regard this as a credible effect modifier: seven covariates were tested in 13 trials, the confidence interval includes no effect, and patient-controlled analgesia is strongly confounded with trial origin, catheter use and background analgesic protocol in this dataset. With 13 trials the ratio of observations to parameters is very low, coefficients are unstable, and R^2^ carries little evidential weight. The defensible summary is that no study-level covariate reliably accounted for the observed heterogeneity, which most plausibly arises from an interplay of technical, pharmacological and institutional factors that study-level regression cannot resolve. Within-trial designs that vary one parameter at a time will be required to answer these questions.

### 3.11. Sensitivity Analyses

**Figure 8:**
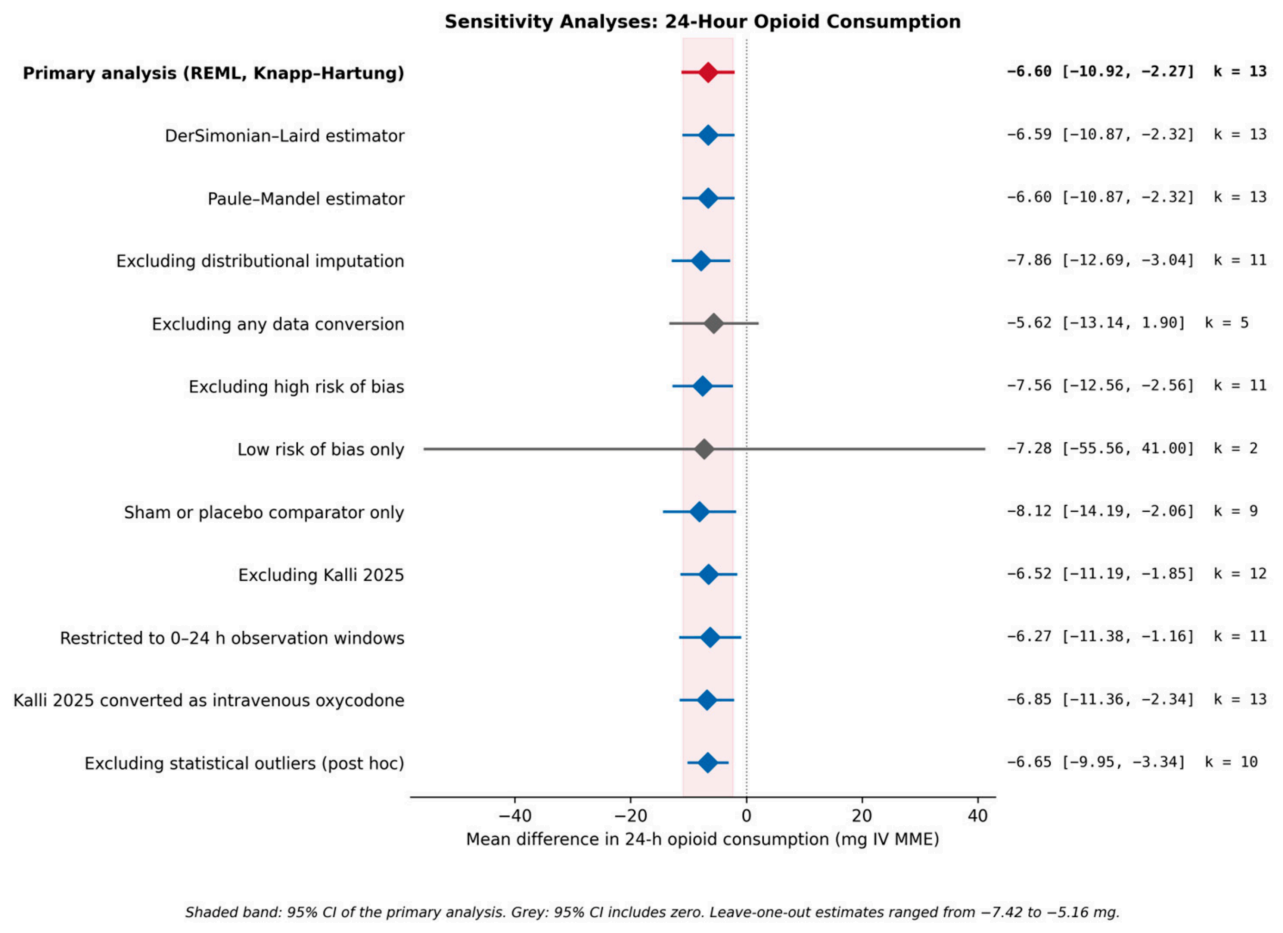
Sensitivity analyses for the primary outcome. The red diamond and shaded band represent the primary estimate and its 95% confidence interval. Blue diamonds represent sensitivity analyses whose 95% confidence interval excludes zero, and grey diamonds those whose interval includes zero. The trial labelled Kalli 2025 is reported in [35,42].

All sensitivity analyses of the primary outcome are reported here and in Figure 8. Re-analysis with the DerSimonian–Laird estimator gave a pooled mean difference of −6.59 mg (95% CI, −10.87 to −2.32), and the Paule–Mandel estimator −6.60 mg (95% CI, −10.87 to −2.32), both essentially identical to the primary analysis. Excluding the two trials whose primary-outcome data required distributional imputation (Pascarella 2023 [37], median-to-mean; Cameron 2025 [47], confidence-interval-to-SD) yielded −7.86 mg (95% CI, −12.69 to −3.04; k = 11), and the stricter variant excluding every trial that required any conversion yielded −5.62 mg (95% CI, −13.14 to 1.90; k = 5), which is of similar magnitude but no longer significant owing to the loss of precision. Restricting the analysis to trials reporting cumulative consumption over 0–24 h, thereby excluding Khera 2021 [11] (48 h) and Abdelaziz 2024 [39] (duration of intensive care stay), gave −6.27 mg (95% CI, −11.38 to −1.16; k = 11). Excluding the two trials at high risk of bias gave −7.56 mg (95% CI, −12.56 to −2.56; k = 11), while restricting to the two trials at low risk of bias gave −7.28 mg with an uninformative confidence interval (−55.56 to 41.00; k = 2); the sham-controlled restriction gave −8.12 mg (95% CI, −14.19 to −2.06; k = 9). Converting the oxycodone consumption reported by Kalli et al. [35,42] as intravenous rather than oral gave −6.85 mg (95% CI, −11.36 to −2.34), and excluding that trial −6.52 mg (95% CI, −11.19 to −1.85). Leave-one-out analysis showed pooled estimates ranging from −7.42 to −5.16 mg, indicating that no single trial drove the result. A post hoc analysis excluding three statistical outliers (Mahrose 2024 [3], Pascarella 2023 [37], Shih 2022 [50]) gave −6.65 mg (95% CI, −9.95 to −3.34; I^2^ = 89.7%). On the standardized scale, the effect was −1.22 (95% CI, −2.16 to −0.27; k = 13) and remained similar when the two trials reporting opioid consumption only over 72 h (Alli 2026 [31]) or 7 days (Cui 2026 [33]) were added (−1.06; 95% CI, −1.89 to −0.23; k = 15). Taken together, the direction of the primary estimate was stable across estimator choice, data handling and risk-of-bias restrictions, whereas its magnitude ranged from about 5 to 8 mg and its statistical significance was lost in the most restrictive analyses.

### 3.12. Certainty of Evidence

The certainty of evidence is summarized in Table 5, and the complete evidence profile, with the judgment and its reason for every domain of every outcome, is provided in Supplementary Material S6. Certainty was very low for all efficacy outcomes except rescue analgesic use, which was rated low. For 24-h opioid consumption, certainty was downgraded for risk of bias (one level; only 16% of the analysis weight came from trials at low risk of bias), inconsistency (two levels; I^2^ = 97.7% with trial estimates on both sides of the null and a prediction interval including an increase in consumption) and publication bias (one level; funnel-plot asymmetry, with the statistical conclusion changing under fixed-effect trim-and-fill). It was not downgraded for indirectness, because population, intervention and comparators matched the review question and restriction to 0–24-h observation windows gave a similar estimate, or for imprecision, because the 95% CI excluded no effect and 902 participants contributed. Pain outcomes were downgraded by two levels for risk of bias, because trials at high risk of bias contributed most of the analysis weight, and additionally for inconsistency, imprecision or both. Time to extubation and ICU length of stay were downgraded for risk of bias and, by two levels, for inconsistency; hospital length of stay additionally for imprecision. Rescue analgesic use was downgraded for risk of bias and imprecision, and PONV for risk of bias, inconsistency and, by two levels, imprecision. The comparison with deep parasternal techniques was rated very low. Certainty for block-related complications was low, downgraded once for risk of bias, reflecting non-systematic ascertainment and incomplete reporting, and once for imprecision, reflecting zero or very few events. Because the minimal clinically important difference was not prespecified, none of these ratings depends on it.

## 4. Discussion

This systematic review and meta-analysis pooled 29 randomized trials, restricted to ultrasound-guided superficial parasternal blocks, to answer a practical question: how much does USG-PIFB actually change the postoperative course after median sternotomy? On average, USG-PIFB reduced 24-h opioid consumption by about 7 mg of intravenous morphine equivalents and lowered pain scores by less than 1.5 points at several time points, and extubation occurred earlier and intensive care stay was shorter. However, heterogeneity was extreme for almost every outcome, the prediction intervals were compatible with no benefit, and the certainty of evidence was very low for nearly all outcomes. Against deep parasternal techniques the difference was close to zero; against the erector spinae plane block the evidence was too sparse to pool. No local anesthetic systemic toxicity or block-attributable pneumothorax was reported, and one block-site hematoma occurred in a USG-PIFB recipient.

For the clinician, the central question is whether a reduction of about 7 mg in 24-h morphine equivalents matters at the bedside, and how reliably it can be expected. The pooled estimate is an average across trials that differed markedly in agent, dose, timing, comparator and background analgesia. With I^2^ close to 98%, the 95% prediction interval (−22.2 to 9.0 mg) indicates that a future trial in a new setting could show a substantial benefit, no benefit or even a small increase in opioid consumption. Both the magnitude and the reproducibility of the opioid-sparing effect therefore remain uncertain, and the pooled mean should not be read as the effect that a given institution can expect. As a descriptive benchmark, the estimate falls below the 10-mg threshold proposed for arthroplasty, which was not prespecified in our registration and has not been validated for cardiac surgery: a smaller absolute reduction might be more valuable after sternotomy, because even modest opioid sparing may facilitate early extubation and deep breathing, whereas a 7-mg reduction distributed across 24 h may be imperceptible to an individual patient. What can be said with more confidence is that USG-PIFB is not a substitute for a multimodal regimen. Within enhanced recovery pathways after cardiac surgery [58], in which chest-wall regional anesthesia is explicitly recommended as one element of multimodal analgesia, a reduction of this size may still contribute usefully alongside acetaminophen, non-steroidal agents, ketamine and dexmedetomidine [59,60].

Pain scores were lower with USG-PIFB both at rest and during movement, but the pooled differences were small (0.8 to 1.4 points), heterogeneity at rest was substantial, trials at high risk of bias contributed most of the analysis weight, and several estimates lost statistical significance when only trials reporting means were analyzed. The data therefore do not support a consistent dissociation between resting and dynamic pain. Anatomically, the superficial parasternal block targets the anterior cutaneous branches of the T2–T6 intercostal nerves, which carry nociceptive input from the sternal periosteum and overlying soft tissue [2,5]. These structures are loaded during coughing, sternal splinting and upper-limb movement, whereas pain after sternotomy also reflects visceral and mediastinal afferents, pericardial irritation and chest-drain sites that lie outside the block’s territory [4,61]. A larger effect on dynamic than on resting pain is therefore plausible, and clinicians evaluating the block at the bedside may reasonably assess it during coughing and mobilization, but this hypothesis requires trials that measure both rest and dynamic pain at standardized time points.

Two features of the evidence base further limit the strength of these conclusions. First, the primary estimate is sensitive to plausible small-study effects: the funnel plot was asymmetric, and although trim-and-fill based on the random-effects model imputed no trials, a fixed-effect implementation moved the estimate close to zero. With I^2^ of 97.7%, asymmetry is at least as likely to reflect genuine clinical and methodological differences between small and large trials as selective publication, and neither implementation provides a corrected estimate; the appropriate reading is that the magnitude of benefit is uncertain, not that it is zero. Second, the exploratory trial sequential analysis did not provide firm evidence in either direction: for anticipated differences of 5 to 10 mg the cumulative z-curve did not cross the monitoring boundaries, and the boundary was crossed only for an anticipated difference of 15 mg, more than twice the pooled estimate. With diversity close to 100%, the required information size is highly sensitive to its assumptions, and we did not base any conclusion on this analysis.

Whether continuous catheter delivery outperforms a single injection remains an open question. Only one trial contributing to the primary outcome used a catheter [52], so no subgroup comparison was possible. The largest trial in this review, a multicentre placebo-controlled trial of catheter-based intermittent boluses, reported a reduction in opioid consumption over 72 h [31], and adding it to the standardized sensitivity analysis did not change the pooled result. Two randomized trials that compared continuous with single-shot delivery directly, and were therefore not eligible for our comparisons, gave conflicting results: catheter boluses after an initial block reduced opioid consumption compared with a placebo infusion in one [30], whereas a continuous infusion did not reduce pain on coughing at 24 h compared with a single injection in the other [62]. Pharmacologically, a benefit from catheters would be plausible—single-shot fascial plane blocks last roughly 8 to 16 h [1,2], whereas post-sternotomy pain persists well beyond 24 h [59,63]—but plausibility is not evidence. Catheters add procedural complexity, infection risk and cost. Until adequately powered trials randomize patients directly to catheter versus single-shot delivery, the choice should rest on local feasibility rather than on a claimed difference in efficacy.

Comparisons with alternative regional techniques are similarly unresolved. Against deep parasternal techniques, six head-to-head comparisons gave a pooled difference near zero with substantial heterogeneity and evenly split effect directions, which is consistent with broadly similar analgesic efficacy but does not demonstrate equivalence. Against the erector spinae plane block only two trials were available, they reported opioid use in non-commensurate formats, and they disagreed in direction. We therefore cannot conclude that the two techniques are comparable. What can reasonably inform practice is the anatomical and safety contrast. Cadaveric work shows that superficial and deep injections produce overlapping but distinct spread [61], and the deep approach passes within millimetres of the internal mammary artery [61,64]. In our safety synthesis no local anesthetic systemic toxicity was reported after USG-PIFB, pneumothorax occurred at similar frequencies in block and control arms and was not attributed to the superficial block, and one block-site hematoma occurred after USG-PIFB; in the three-arm trial that reported pneumothorax by arm, it was more frequent after the deep block [45]. These observations are reassuring, but given rare events, incomplete reporting and a report of suspected systemic toxicity during continuous superficial parasternal infusion in a trial outside our eligibility criteria [62], they do not establish that USG-PIFB is safer than its alternatives. Where both approaches are feasible, the superficial technique remains attractive on anatomical grounds rather than on demonstrated superiority.

Several limitations qualify these findings. Statistical heterogeneity remained extreme for most outcomes despite restricting inclusion to ultrasound-guided techniques, reflecting diversity in agent, dose, adjuvant, timing, number of injection levels and background analgesia; our meta-regression could not resolve it, and with 13 trials those analyses are vulnerable to overfitting and are reported as exploratory only. Most trials were small and single-centre, only 4 of 29 were at overall low risk of bias and 12 were at high risk, most often because of absent sham injection, asymmetric co-interventions or concerns across several domains. Several outcomes required median-to-mean conversion, two trials contributed opioid data over windows other than 24 h, and opioid doses were harmonized with equianalgesic ratios that are themselves approximate. Several analytic elements, including the designation of the primary outcome, the minimal clinically important difference thresholds, the trial sequential analysis, trim-and-fill and the dose–response analysis, were not specified in the original registration (Supplementary Material S1, Table S1). The search update was limited to openly accessible sources, so recent trials indexed only in Embase, CENTRAL, CINAHL, Web of Science or Scopus may have been missed. The dose–response analysis is ecological and rests on 11 trials, although its null result persisted within agent-specific strata, so it is not simply an artefact of pooling non-equipotent drugs. Head-to-head evidence is sparse. Finally, few trials assessed chronic post-sternotomy pain, patient-reported quality of recovery or cost-effectiveness—omissions that matter, since chronic post-sternotomy pain affects an estimated 10–40% of patients [59,63,65,66] and poorly controlled acute pain is among its strongest predictors [67,68].

Priorities for future research follow directly. Large multicentre sham-controlled trials with standardized outcome reporting are needed, ideally reporting opioid consumption in intravenous morphine milligram equivalents over fixed windows, pain at rest and on movement at fixed time points, and block-related complications for each arm, since non-commensurate reporting was the single greatest obstacle to synthesis in this review. Within-trial dose-finding designs that vary concentration and volume while holding technique constant would answer the dose question that study-level regression cannot. Adequately powered trials randomizing patients directly to catheter versus single-shot delivery would settle the question that the available evidence can only raise. Head-to-head trials against the erector spinae plane block, reporting commensurate opioid outcomes, are required before any claim of equivalence is made. Finally, chronic pain at 3 and 12 months, quality of recovery and health economic endpoints should be incorporated as secondary outcomes.

## 5. Conclusions

This systematic review and meta-analysis provides very low certainty evidence that ultrasound-guided superficial pectointercostal fascial block reduces 24-h opioid consumption after cardiac surgery via median sternotomy by a modest amount on average, with small reductions in pain scores. Because heterogeneity was extreme and the prediction interval included no effect, both the magnitude and the reproducibility of the opioid-sparing effect across clinical settings remain uncertain, and the estimate is sensitive to plausible small-study effects. No local anesthetic systemic toxicity or block-attributable pneumothorax was reported and one block-site hematoma occurred in a USG-PIFB recipient, but the available data are insufficient to claim a safety advantage over alternative techniques. Whether continuous catheter delivery is superior to single-shot injection, and whether USG-PIFB is equivalent to deep parasternal or erector spinae plane blocks, cannot be determined from the present evidence and should not be inferred from the absence of a demonstrated difference. USG-PIFB is best regarded as one component of multimodal analgesia within enhanced recovery pathways after cardiac surgery rather than as a standalone technique. Adequately powered, sham-controlled multicentre trials with standardized outcome reporting remain the principal research priority.

## Figures and Tables

**Table 1:** Characteristics of included randomized controlled trials. N, number of patients (randomized or analyzed, as reported); I/C, intervention/control; LA, local anesthetic; Conc., concentration; Vol., total bilateral volume (mL); Cath., continuous catheter; S-PIP, superficial pectointercostal plane; ESPB, erector spinae plane block; TTMPB, transversus thoracic muscle plane block; D-PIP, deep parasternal intercostal plane block; CPB, cardiopulmonary bypass; OPCAB, off-pump coronary artery bypass; PCIA, patient-controlled intravenous analgesia; NR, not reported. Age is reported as mean ± SD or median (IQR) for the intervention (block) group, and male sex for the intervention group, unless stated otherwise. All blocks were bilateral. Three studies in Comparison 1 (Alli 2026 [31], Zhang 2022 [52], Zhao 2025 [10]) employed catheter delivery; all other studies used single-shot injection. Five head-to-head studies (Comparison 2) lacked a no-block/sham control arm. Three multi-arm studies (Demarquette 2025 [45], Toker 2025 [56], Yadav 2024 [43]) contributed data to both Comparison 1 (S-PIP vs. control arm) and Comparison 2 (S-PIP vs. alternative block arm).

Study	Country	N (I/C)	Age (y)	Male	Surgery Type	Timing	LA Agent	Conc.	Vol.	Adjuvant	Cath.	Comparator
Comparison 1: USG-Guided S-PIP Block versus Control (k = 24)												
Abdelaziz (2024) [39]	Egypt	40 (20/20)	57.2 ± 8.1	10 (50%)	CABG only (on-pump; 2–3 vessel)	Post-induction, pre-incision	Bupivacaine + Lidocaine (mixture)	0.5%	40	None	No	Sham (saline)
Alli (2026) [31]	Canada (4 centres)	340 (164/154)	68 (61–73)	122 (74.4%)	Mixed (CABG 71%, valve 9%, CABG + valve 7%, other 13%)	Post-closure (catheter, 48 h)	Ropivacaine	0.2%	40	None	Yes	Placebo (saline via identical catheters)
Cameron (2025) [47]	Canada	60 (28/32)	NR	NR	CABG only (on-pump; 2–5 vessels)	Post-incision (end of surgery)	Bupivacaine	0.25%	60	Dexamethasone, dexmedetomidine, epinephrine	No	No block (identical dressings only)
Cui (2026) [33]	China	284 (126/134)	67.1 ± 5.2	92 (73.0%)	OPCAB only (age ≥ 60 years)	Post-intubation	Ropivacaine	0.375%	40	None	No	Placebo (saline PIFB); 2 × 2 factorial design
Demarquette (2025) [45]	France	254 (84/85)	64.3 ± 10.0	70 (83.3%)	Valve, CABG, Valve + CABG, Aorta, Other (all sternotomy)	Post-closure	Ropivacaine	0.2%	40	None	No	No block (standard care)
Eloraby (2024) [48]	Egypt	90 (30/30)	46.0 ± 10.0	15 (50%)	Valve replacement only (MVR, AVR, MVR + TVR, double MVR + AVR)	Post-incision (end of surgery, after skin closure)	Bupivacaine	0.25%	40	Dexamethasone	No	No block (systemic analgesia only)
Fadhlurrahman (2024) [40]	Indonesia	40 (20/20)	52.5	18 (90%)	Open heart surgery with sternotomy and CPB (specific types not listed)	Pre-incision (preemptive)	Ropivacaine	0.375%	40	None	No	No block (opioid-based analgesia alone)
Hamed (2022) [41]	Egypt	70 (35/35)	53.9 ± 9.9	28 (80%)	On-pump CABG, MVR, AVR, ASD, CABG + MVR (sternotomy)	Post-closure	Bupivacaine	0.25%	40	Epinephrine 5 µg/mL	No	Sham (dry needling)
Huette (2026) [34]	France	82 (41/41)	65.1 ± 8.8 (all)	56 (68.3%, all)	Elective cardiac surgery with CPB	Post-induction, pre-incision	Ropivacaine	0.2%	40	None	No	No block (standard multimodal analgesia)
Kalli (2025) [42]	Finland	74 (37/37)	66.7 ± 10.7	NR	Surgical aortic valve replacement (isolated AVR via full sternotomy)	Pre-incision	Ropivacaine	0.75%	40	None	No	Sham (saline injection)
Khera (2021) [11]	USA	80 (40/40)	65.8 ± 8.7	27 (67.5%)	CABG 55–65%, CABG + valve 7.5–12.5%, Valve 20–35%, Other 2.5%	Post-operative (POD0 within 2 h of ICU admission + repeated POD1)	Bupivacaine	0.25%	40	None	No	Placebo (saline PIFB, 20 mL per side, two doses)
Kumar (2021) [44]	India	40 (20/20)	51.2 ± 15.9	11 (55.0%)	Mixed cardiac surgery (CABG + valvular + others) on CPB	Post-closure	Ropivacaine	0.25%	60	None	No	No block
Mahrose (2024) [3]	Egypt	40 (20/20)	54.1 ± 8.4	15 (75%)	OPCAB only (off-pump CABG)	Pre-incisional (after induction, before surgery)	Bupivacaine + Adrenaline	0.25%	40	Adrenaline 2.5 mcg/mL	No	Sham (saline block); drain-site infiltration in both groups
Malik (2026) [32]	India	85 (50/25)	NR	NR	Elective cardiac surgery via median sternotomy	Pre-incision	Ropivacaine	0.4%	40	Fentanyl 25 or 50 µg per side	No	No block
Pascarella (2023) [37]	Italy	126 (63/63)	67.0 ± 10.0	33 (52%)	Mixed cardiac (CABG 57–65%, valvular 25–30%, off-pump, combined, aortic aneurysm)	Pre-incision	Ropivacaine	0.5%	40	None	No	No block; drain-site infiltration in both groups
Powers (2025) [49]	India	90 (45/45)	63.7 ± 4.2	NR	CABG only (on-pump, elective)	Pre-incision	Levobupivacaine	0.5%	30	None	No	Active non-regional: IV morphine infusion (control arm only)
Shih (2022) [50]	USA	52 (27/25)	71	21 (77.8%)	Mixed cardiac surgery via full sternotomy (CABG 60–67%, AVR 4–7%, CABG + AVR 18–25%, Other 7–12%)	Pre-incisional	Bupivacaine (liposomal + plain)	1.3%	50	None	No	Normal saline injection (volume-matched sham)
Singh (2024) [38]	India	70 (23/22)	41.3 ± 11.5	12 (60%)	Mixed cardiac (valve 45–70%, CABG 20–35%, septal defect repair 10–25%)	Postoperative (1 h before extubation, while intubated in ICU)	Bupivacaine (plain)	0.125%	30	None (Group B)	No	No block
Toker (2025) [56]	Türkiye	75 (25/25)	63.4 ± 6.4	11 (44%)	Isolated CABG only (elective)	Post-incision (after sternal closure and sterile prep)	Bupivacaine	0.375%	60	None	No	No block; drain-site infiltration in all groups
Wang (2023) [51]	China	50 (25/25)	62.2 ± 12.9	16 (64%)	Mixed cardiac (CABG 76%, valve 6%, CABG + valve 18%)	Pre-incision (after GA induction, before surgery)	Ropivacaine	0.3%	60	Dexamethasone	No	Placebo (saline, 30 mL per side)
Wang (2025) [46]	China	110 (55/55)	67.3 ± 5.2	36 (65.5%)	Off-pump CABG	Pre-incision	Ropivacaine	0.4%	40	None	No	Sham (saline)
Yadav (2024) [43]	India	60 (20/20)	41.1 ± 10.7	9 (45.0%)	Mixed cardiac (CABG 20%, MVR 30%, AVR 20%, ASD 10%, VSD 20% in Group P)	Pre-incision	Ropivacaine	0.2%	60	None	No	No block
Zhang (2022) [52]	China	110 (55/55)	48.7 ± 17.5	26 (47.3%)	Valve surgery only (MVR or AVR)	Pre-incision	Ropivacaine	0.33%	40	None	Yes	Sham (saline)
Zhao (2025) [10]	China	112 (37/38)	56.9 ± 10.8	17 (45.9%)	Mixed cardiac (single/double/triple valvuloplasty or replacement + congenital heart disease)	Pre-incision	Ropivacaine	0.3%	40	None	Yes	Active non-regional: PCIA (sufentanil, esketamine, dexmedetomidine)
***Comparison 2: USG-Guided S-PIP Block versus Alternative Regional Technique (k = 5 head-to-head trials)***												
Elbardan (2024) [55]	Egypt	100 (50/50)	Median: 59	29 (58%)	CABG, MVR, AVR (all on-pump, sternotomy)	Pre-incision	Bupivacaine	0.25%	60	None	No	ESPB
Kaya (2022) [53]	Türkiye	42 (21/21)	62	14 (73.7%)	CABG, AVR, MVR, AVR + MVR (median sternotomy)	Pre-incision	Bupivacaine	0.25%	40	Epinephrine	No	TTMPB
Keshwani (2023) [57]	India	38 (19/19)	36.6 ± 10.8	9 (60%)	Elective cardiac surgery via midline sternotomy (mixed—valve, CABG, vascular repair)	Post-closure	Ropivacaine	0.2%	40	None	No	ESPB
Mansour (2024) [54]	Egypt	80 (40/40)	52.7 ± 15.8	29 (72.5%)	Mixed on-pump cardiac surgery via sternotomy	Pre-incisional (after intubation, before incision)	Bupivacaine (both groups)	0.25%	40	None	No	TTMPB
Vanjare (2025) [36]	India	118 (59/59)	55.9 ± 8.3	NR	Cardiac surgery via sternotomy (CAD 61%, valvular 39%)	Post-induction	Ropivacaine	0.2%	40	None	No	TTMPB

**Table 2:** Pooled pain score analyses at rest and during movement. MD, mean difference on the 0–10 NRS (negative values favor USG-PIFB); k, number of studies; PI, prediction interval (not estimated when k < 3). The last column shows the analysis restricted to trials reporting means and standard deviations (not estimated when fewer than two such trials contributed).

Time Point	Condition	k	MD (95% CI)	*p*	I^2^ (%)	95% PI	Means and SDs Only: MD (95% CI) [k]
0 h (Extubation)	Rest	7	−1.38 (−2.31 to −0.45)	0.011	97.0	−4.05 to 1.28	−1.51 (−4.84 to 1.82) [3]
6 h	Rest	5	−1.08 (−2.48 to 0.32)	0.100	93.4	−4.85 to 2.70	—
12 h	Rest	7	−0.84 (−1.39 to −0.28)	0.010	79.1	−2.32 to 0.65	−0.81 (−1.54 to −0.08) [3]
24 h	Rest	12	−0.90 (−1.54 to −0.26)	0.010	96.5	−3.10 to 1.31	−1.12 (−2.49 to 0.24) [6]
0 h (Extubation)	Movement	3	−0.75 (−1.94 to 0.45)	0.115	80.2	−7.18 to 5.69	—
6 h	Movement	2	−1.79 (−4.31 to 0.73)	0.070	0.0	—	—
12 h	Movement	4	−1.29 (−1.81 to −0.77)	0.004	0.0	−1.99 to −0.59	−1.23 (−2.03 to −0.43) [3]
24 h	Movement	6	−1.01 (−2.05 to 0.04)	0.056	92.4	−3.81 to 1.79	−0.78 (−4.16 to 2.61) [3]

**Table 3:** Subgroup analyses for 24-h opioid consumption. MD, mean difference in mg intravenous morphine milligram equivalents (negative values favour USG-PIFB); k, number of trials. All subgroup estimates use the same random-effects model as the primary analysis (REML with Knapp–Hartung adjustment); for the single catheter trial, the trial estimate is shown and no interaction test was performed. Neither of the two interaction tests was statistically significant, and all subgroup analyses are exploratory and hypothesis-generating.

Subgroup	k	MD (95% CI), mg	I^2^ (%)	*p* (Interaction)
Overall	13	−6.60 (−10.92 to −2.27)	97.7	—
Delivery method				Not performed
Single-shot injection	12	−6.11 (−10.74 to −1.48)	97.2	
Continuous catheter	1	−12.00 (−13.54 to −10.46)	—	
Comparator type				0.17
Sham/placebo	9	−8.12 (−14.19 to −2.06)	93.8	
No block	4	−3.36 (−10.67 to 3.94)	93.2	
Block timing				0.07
Preoperative	9	−7.89 (−14.04 to −1.73)	98.5	
Postoperative	4	−2.69 (−5.91 to 0.54)	22.0	

**Table 4:** Univariate meta-regression of potential moderators of 24-h opioid consumption. Slope is the meta-regression coefficient from a random-effects model with REML estimation and Knapp–Hartung adjustment; τ^2^, residual between-study variance; I^2^ residual, residual heterogeneity; R^2^, proportion of between-study variance explained (descriptive). All analyses are univariate and exploratory; no covariate is claimed as an effect modifier. Binary covariates with fewer than two trials in either category were not estimable.

Covariate	k	Slope (95% CI)	τ^2^ (res.)	I^2^ res. (%)	R^2^ (%)	*p*-Value
Total LA dose (mg)	11	0.047 (−0.037 to 0.130)	42.0	94.4	9.2	0.236
Total volume (mL)	13	0.307 (−0.149 to 0.763)	42.3	97.9	9.1	0.167
LA concentration (%)	11	7.694 (−27.609 to 42.997)	49.4	95.5	0.0	0.634
α2-agonist adjuvant	13	Not estimable	—	—	—	—
Dexamethasone adjuvant	13	7.006 (−4.626 to 18.639)	43.6	97.9	6.3	0.212
Preoperative block timing	13	−4.409 (−13.935 to 5.118)	45.8	97.9	1.7	0.330
Delivery mode (catheter vs. single-shot)	13	Not estimable	—	—	—	—
Sham vs. no-block comparator	13	−4.715 (−13.912 to 4.481)	45.2	93.6	2.9	0.283
Patient-controlled analgesia used	13	−7.833 (−16.019 to 0.353)	34.7	96.4	25.4	0.059

**Table 5:** GRADE summary of findings. Certainty was rated in a non-null effect using the explicit domain criteria in Section 2.7.7. MD, mean difference; OR, odds ratio; MME, morphine milligram equivalents; CI, confidence interval; ICU, intensive care unit; LAST, local anesthetic systemic toxicity; k, number of contributing trials; N, number of contributing participants. Downgraded domains are shown with the number of levels in parentheses; domains not listed were not downgraded, and the reasons for every judgment are given in Supplementary Material S6. Certainty symbols: ⊕⊕⊕⊕ high; ⊕⊕⊕○ moderate; ⊕⊕○○ low; ⊕○○○ very low.

Outcome	k	N	Effect (95% CI)	Domains Downgraded (Levels)	Certainty
24-h opioid consumption (mg IV MME)	13	902	MD −6.60 (−10.92 to −2.27)	Risk of bias (−1); inconsistency (−2); publication bias (−1)	⊕○○○ Very low
Pain at rest, 24 h (NRS 0–10)	12	880	MD −0.90 (−1.54 to −0.26)	Risk of bias (−2); inconsistency (−2)	⊕○○○ Very low
Pain during movement, 24 h (NRS 0–10)	6	417	MD −1.01 (−2.05 to 0.04)	Risk of bias (−2); inconsistency (−2); imprecision (−1)	⊕○○○ Very low
Time to extubation	16	1563	MD −106.1 min (−174.4 to −37.9)	Risk of bias (−1); inconsistency (−2)	⊕○○○ Very low
ICU length of stay	14	1496	MD −13.4 h (−21.3 to −5.4)	Risk of bias (−1); inconsistency (−2)	⊕○○○ Very low
Hospital length of stay	10	1116	MD −0.66 days (−1.51 to 0.19)	Risk of bias (−1); inconsistency (−2); imprecision (−1)	⊕○○○ Very low
Rescue analgesic use	6	596	OR 0.61 (0.32 to 1.15)	Risk of bias (−1); imprecision (−1)	⊕⊕○○ Low
Postoperative nausea and vomiting	7	720	OR 0.62 (0.24 to 1.61)	Risk of bias (−1); inconsistency (−1); imprecision (−2)	⊕○○○ Very low
Comparison 2: USG-PIFB vs. deep parasternal blocks, 24-h opioid consumption (mg IV MME)	6	496	MD 0.43 (−3.93 to 4.79)	Risk of bias (−1); inconsistency (−2); imprecision (−1)	⊕○○○ Very low
Local anesthetic systemic toxicity	16	1215	Events, USG-PIFB vs. comparator: 0/606 vs. 0/609	Risk of bias (−1); imprecision (−1)	⊕⊕○○ Low
Block-site hematoma	17	1255	Events, USG-PIFB vs. comparator: 1/626 vs 3/629	Risk of bias (−1); imprecision (−1)	⊕⊕○○ Low
Pneumothorax (none attributed to the block)	17	1475	Events, USG-PIFB vs. comparator: 8/732 vs. 8/743	Risk of bias (−1); imprecision (−1)	⊕⊕○○ Low

## Data Availability

All data supporting the findings of this study are contained within the article and its Supplementary Materials. The complete analysis code, the study-level analytical dataset, the opioid conversion table, the data-conversion log, the search strategies and screening log, the domain-level RoB 2 judgments and the complete GRADE evidence profile are provided as Supplementary Materials S1, S3, S5 and S6, enabling independent reproduction of every analysis reported here.

## Supplementary Materials

The following supporting information can be downloaded at: https://www.mdpi.com/article/10.3390/medicina62101890/s1. Supplementary Material S1: deviations from the registered protocol (Table S1); Supplementary Material S2: study-level contribution map and comparator classification (Table S2a,b); Supplementary Material S3: search strategies for the original and update searches, update screening log and registered trials without results (Table S3a–e); Supplementary Material S4: analysis code, study-level analytical dataset, opioid conversion table and data-conversion log; Supplementary Material S5: domain-level RoB 2 judgments, support statements, overall-judgment rationale and consensus log (Table S5a–d); Supplementary Material S6: GRADE evidence profile (Table S6a) and exploratory trial sequential analysis (Table S6b, Figure S1); Supplementary Material S7: study-level adverse-event table (Table S7); Supplementary Material S8: completed PRISMA 2020 checklist.
