# Supplementary material for: Efficacy of Ultrasound-Guided Pectointercostal Fascial Block Versus Control and Alternative Regional Techniques in Cardiac Surgery: A Systematic Review and Meta-Analysis with Dose–Response Analysis
Source: Medicina (Kaunas). 2026 Sep 29;62(10):1890. doi: 10.3390/medicina62101890 (PMC13707331; doi:10.3390/medicina62101890)
Supplement: Supplementary file 1 [file medicina-62-01890-s001.zip › Supplementary_Materials_S1_S2_S5_S6_S7.pdf]

Supplementary Materials

Efficacy of Ultrasound-Guided Pectointercostal Fascial Block Versus Control and Alternative Regional Techniques in Cardiac Surgery: A Systematic Review and Meta-Analysis with Dose–Response Analysis

Supplementary Material S1. Deviations from the registered protocol (Table S1)

Table S1 lists the differences between the original PROSPERO registration (version 1.0, registered on 5 August 2025, before the literature search) and the review as conducted and reported.

Table S1. Deviations from the registered protocol (PROSPERO CRD420251114732, version 1.0). The retrospective amendment (version 2.0, 9 March 2026) was submitted after the analyses had been completed; elements introduced during the conduct of the review are therefore post hoc relative to the original registration. GRADE, Grading of Recommendations, Assessment, Development and Evaluations; MCID, minimal clinically important difference; NRS, numeric rating scale; PIFB, pectointercostal fascial block; REML, restricted maximum likelihood; RoB 2, Cochrane risk-of-bias tool for randomized trials, version 2.

Item	Original registration (version 1.0)	Review as conducted	Timing and rationale
Primary outcome	Five main outcomes listed (pain intensity, 24-h opioid consumption, time to first rescue analgesia, ventilation and recovery times, analgesia-related complications); pain intensity listed first; no outcome designated as primary	24-h opioid consumption (IV MME) designated as the sole primary outcome; pain intensity analyzed as a secondary outcome	Changed during the review because opioid consumption was the most consistently reported endpoint; recorded in version 2.0
Pain time points	6, 12, 24 and 48 h after surgery	Extubation, 6, 12 and 24 h, at rest and during movement; 48 h not analyzed	Aligned with the time points reported by the trials; recorded in version 2.0
Other outcomes	Time to first rescue analgesia; respiratory events; additional outcomes: quality of recovery, satisfaction, chronic pain, block performance, inflammatory markers, costs	Rescue analgesic use, extubation, ICU and hospital stay, PONV and block-related complications synthesized; time to first rescue analgesia summarized narratively; additional outcomes not synthesized	Insufficient or non-commensurate reporting
Study designs	Randomized and quasi-randomized trials; non-randomized controlled studies and prospective cohorts for qualitative synthesis	Randomized controlled trials only	Sufficient randomized evidence; recorded in version 2.0
Intervention	Ultrasound-guided PIFB with any local anesthetic, 15–30 mL, with or without adjuvants	Ultrasound-guided superficial block only (single-shot or catheter), any concentration, 30–60 mL in total; surgeon-performed, landmark-based and deep blocks without a superficial arm excluded	Clarification of eligibility during screening; recorded in version 2.0
Comparators	Sham or placebo injection, no regional block, or ultrasound-guided ESPB/TTPB; systemic analgesia-only arms without a sham procedure excluded	Comparison 1: sham/placebo, no block or active non-regional comparator; Comparison 2: ESPB or D-PIP/TTMPB	No-block controls were included; their influence was examined in subgroup and sensitivity analyses (comparator classification in Table S2b)
Information sources	CENTRAL, CINAHL, Embase, MEDLINE, SSCI and Scopus; contact with authors, reference lists and trial registers; published studies only	Web of Science Core Collection searched; registers, preprint servers and conference proceedings screened; unpublished trials sought; update search of openly accessible sources and registers to 17 September 2026	Broadened to identify unpublished and recent trials (version 2.0); the update search was limited to openly accessible sources
Meta-analysis model	DerSimonian–Laird random effects; RevMan and/or R	REML with ad hoc Knapp–Hartung adjustment; DerSimonian–Laird and Paule–Mandel estimators in sensitivity analyses; Python	More conservative inference with few trials; recorded in version 2.0
Dichotomous effect measure	Risk ratio	Odds ratio	Recorded in version 2.0

Item	Original registration (version 1.0)	Review as conducted	Timing and rationale
Subgroup analyses	Block technique variant, use of adjuvants, type of surgery	Delivery method, comparator type, block timing; adjuvants examined by meta-regression; type of surgery not analyzed	Recorded in version 2.0; post hoc relative to version 1.0
Sensitivity analyses	Exclusion of studies at high risk of bias; alternative effect models	Also leave-one-out, low risk of bias only, sham-controlled only and exclusion of imputed or converted data (version 2.0); 0–24-h windows, oxycodone conversion, standardized mean difference with longer windows, outlier exclusion and means-only analyses (post hoc)	Labelled as post hoc in Section 2.7.3
Additional analyses	Not planned	Meta-regression (nine covariates) with dose–response analysis; prediction intervals; trial sequential analysis; trim-and-fill; MCID thresholds (10 mg; 1.0 NRS point)	Added in version 2.0, after the analyses; MCID thresholds used descriptively only; trial sequential analysis exploratory
Risk of bias	RoB 2 and ROBINS-I	RoB 2 only, with the full RoB 2 overall-judgment rules	No non-randomized studies included
Certainty of evidence	GRADE; domain criteria not specified (version 2.0: imprecision judged against MCID thresholds)	GRADE with explicit criteria for every domain; certainty rated in a non-null effect, independent of MCID thresholds	Post hoc; the MCID thresholds were not part of the original registration and have not been validated for cardiac surgery

Supplementary Material S2. Study-level contribution map and comparator classification (Tables S2a and S2b)

For each of the 29 included randomized controlled trials, Table S2a shows the comparison(s) to which the trial contributed, the classification of its comparator, and the analyses in which it was included. C1, Comparison 1 (USG-PIFB versus sham or placebo injection, no block, or an active non-regional comparator); C2, Comparison 2 (USG-PIFB versus an alternative regional technique). Comparators were classified as sham/placebo (an injection or procedure without active local anesthetic), no block (standard systemic analgesia without a sham procedure) or active non-regional (an analgesic intervention given to the control arm only). Co-interventions delivered identically to both arms did not change the classification. Three multi-arm trials (Demarquette 2025, Toker 2025, Yadav 2024) contributed a USG-PIFB-versus-control comparison to C1 and a USG-PIFB-versus-alternative-block comparison to C2; their shared USG-PIFB arm was never counted twice within a single analysis. Y, contributed; –, not reported in a form permitting synthesis. Table S2b gives, for each of the 13 trials contributing to the primary outcome, the control arm as described in the trial report, the co-interventions delivered to both arms, the comparator classification, the observation window of the opioid data and any data transformation applied.

Table S2a. Study-level contribution map. Participants are given as USG-PIFB/comparator arm as analyzed or reported. Pain time points: ext, extubation; 6, 12 and 24, hours. SMD, standardized mean difference; ESPB, erector spinae plane block; D-PIP, deep parasternal intercostal plane block; TTMPB, transversus thoracic muscle plane block; TTPB, transversus thoracis plane block.

Trial	Comparison	Participants (USG-PIFB/comparator)	Comparator classification	24-h opioid consumption	Pain at rest (time points)	Pain on movement (time points)	Extubation	ICU stay	Hospital stay	Rescue analgesia	PONV	Block-related complications reported	Overall RoB 2
Abdelaziz 2024	C1	20/20	Sham/placebo	Y	6	–	Y	Y	–	–	–	Y	Some concerns
Alli 2026	C1	164/154	Sham/placebo	72 h (SMD sensitivity)	–	–	Y	Y	Y	–	Y	Serious events only	Low
Cameron 2025	C1	28/32	No block	Y	24	24	–	–	–	–	–	Y	Some concerns
Cui 2026	C1	126/134	Sham/placebo	7 days (SMD sensitivity)	–	–	Y	Y	–	Y	–	Y	Some concerns
Demarquette 2025	C1 + C2	84/85 (+85 D-PIP)	No block; D-PIP (C2)	Y (C2)	–	–	–	Y	Y	–	–	Y	Some concerns
Elbardan 2024	C2	50/50	ESPB	–	–	–	–	–	–	–	–	Y	Low
Eloraby 2024	C1	30/30	No block	–	ext, 6, 12, 24	–	Y	–	–	–	–	–	High
Fadhlurrahman 2024	C1	20/20	No block	–	–	–	Y	Y	–	–	–	–	High
Hamed 2022	C1	35/35	Sham/placebo	Y	–	–	Y	Y	Y	–	–	Y	Low
Huette 2026	C1	41/41	No block	–	24	24	Y	Y	Y	–	–	Y	High
Kalli 2025	C1	37/37	Sham/placebo	Y	–	–	–	–	–	–	–	–	Some concerns
Kaya 2022	C2	21/21	TTMPB	Y	–	–	–	–	–	–	–	Y	Some concerns
Keshwani 2023	C2	19/19	ESPB	–	–	–	–	–	–	–	–	–	High
Khera 2021	C1	40/40	Sham/placebo	Y	–	–	–	Y	Y	Y	–	Y	Some concerns
Kumar 2021	C1	20/20	No block	–	ext, 6, 12	ext, 6, 12	Y	–	–	–	–	Y	High
Mahrose 2024	C1	20/20	Sham/placebo	Y	–	–	Y	Y	–	Y	Y	Y	Some concerns
Malik 2026	C1	50/25	No block	–	ext, 24	–	Y	Y	Y	–	Y	–	High

Trial	Comparison	Participants (USG-PIFB/comparator)	Comparator classification	24-h opioid consumption	Pain at rest (time points)	Pain on movement (time points)	Extubation	ICU stay	Hospital stay	Rescue analgesia	PONV	Block-related complications reported	Overall RoB 2
Mansour 2024	C2	40/40	TTMPB	Y	–	–	–	–	–	–	–	–	High
Pascarella 2023	C1	63/63	No block	Y	ext, 6, 12, 24	–	Y	–	–	Y	–	–	High
Powers 2025	C1	45/45	Active non-regional	–	–	–	Y	–	–	–	–	Y	High
Shih 2022	C1	27/25	Sham/placebo	Y	24	–	Y	Y	Y	–	Y	Y	Some concerns
Singh 2024	C1	23/22	No block	–	6, 12, 24	6, 12, 24	–	–	–	Y	–	Y	High
Toker 2025	C1 + C2	25/25 (+25 D-PIP)	No block; D-PIP (surgeon-performed) (C2)	Y; Y (C2)	ext, 12, 24	ext, 24	–	–	–	–	–	Y	High
Vanjare 2025	C2	59/59	TTMPB	Y	–	–	–	–	–	–	–	–	Some concerns
Wang 2023	C1	25/25	Sham/placebo	Y	–	12	Y	Y	Y	Y	Y	–	Some concerns
Wang X 2025	C1	55/55	Sham/placebo	Y	ext, 12, 24	–	Y	Y	Y	–	–	Y	Low
Yadav 2024	C1 + C2	20/20 (+20 TTPB)	No block; TTPB (C2)	Y; Y (C2)	24	–	–	–	–	–	–	–	Some concerns
Zhang 2022	C1	55/55	Sham/placebo	Y	24	24	Y	Y	Y	–	Y	Y	Some concerns
Zhao 2025	C1	37/38	Active non-regional	–	ext, 12, 24	ext, 12, 24	–	–	–	–	Y	Y	High

Totals: 29 unique trials; 24 contributed to Comparison 1 and 8 to Comparison 2 (5 dedicated head-to-head trials and 3 multi-arm trials). Across the 29 trials, 2,812 patients were randomized and 2,664 contributed data to this review (2,156 in the Comparison 1 arms, 378 in the five dedicated head-to-head trials and 130 in the alternative-block arms of the multi-arm trials); the remaining 148 were randomized to trial arms that did not meet the eligibility criteria or were excluded from the analyzed populations of the trials. Two trials (Alli 2026, Cui 2026) reported opioid consumption only over 72 hours or 7 days and contributed to the standardized mean difference sensitivity analysis.

Table S2b. Comparator classification of the 13 trials contributing to the primary outcome (24-hour opioid consumption). Co-interventions delivered identically to both arms did not change the classification (Section 2.2.3 of the main text). ICU, intensive care unit; IQR, interquartile range; MME, morphine milligram equivalents; PCA, patient-controlled analgesia; PCIA, patient-controlled intravenous analgesia; PIFB, pectointercostal fascial block; SD, standard deviation.

Trial	Control arm	Co-interventions in both arms	Classification	Opioid outcome window	Data transformation
Abdelaziz 2024	Sham block with 1 mL saline per side (not volume-matched)	Paracetamol and ketorolac; morphine as needed	Sham/placebo	Duration of ICU stay (mean 27 h vs 70 h)	None
Cameron 2025	No injection; identical dressings	Acetaminophen; hydromorphone, ketorolac and ketamine as needed; dexmedetomidine sedation	No block	0–24 h	SD from 95% CI; hydromorphone ×5
Hamed 2022	Dry needling without injectate	Provider-administered morphine in the ICU	Sham/placebo	24 h after extubation	None
Kalli 2025	Saline, 40 mL (same technique)	Oxycodone (oral and intravenous)	Sham/placebo	0–24 h	Oxycodone ×0.5 (×1.0 in sensitivity analysis)
Khera 2021	Saline PIFB, 20 mL per side, on two days	Physician-directed analgesia (institutional standard of care)	Sham/placebo	0–48 h (24-h data not reported)	None
Mahrose 2024	Saline block, 20 mL per side	Drain-site bupivacaine infiltration; paracetamol; fentanyl PCA	Sham/placebo	0–24 h	Fentanyl ×0.1
Pascarella 2023	No injection	Drain-site ropivacaine infiltration; morphine as needed	No block	0–24 h	Median (IQR) to mean (SD)

Trial	Control arm	Co-interventions in both arms	Classification	Opioid outcome window	Data transformation
Shih 2022	Saline injection	Team-directed multimodal analgesia (ketorolac; opioids as needed)	Sham/placebo	0–24 h	None (reported as MME)
Toker 2025	No parasternal injection	Drain-site bupivacaine infiltration; paracetamol; nurse-initiated tramadol	No block	0–24 h	Tramadol ×0.1
Wang 2023	Saline, 30 mL per side	Hydromorphone PCIA	Sham/placebo	0–24 h	Hydromorphone ×5
Wang X 2025	Saline injection (same technique)	Sufentanil PCIA	Sham/placebo	0–24 h	None (reported as morphine equivalents)
Yadav 2024	No block	Paracetamol; fentanyl infusion until extubation and rescue boluses	No block	0–24 h	Fentanyl ×0.1
Zhang 2022	Saline bolus and saline catheter infusion	Sufentanil PCIA; flurbiprofen on request	Sham/placebo	Postoperative day 1	Sufentanil ×0.5

Supplementary Material S5. Risk of bias assessment (RoB 2) (Tables S5a–S5d)

Risk of bias was assessed with the Cochrane RoB 2 tool (effect of assignment) for the outcomes of each trial. For every domain, a support statement documenting the relevant trial evidence was recorded, and two assessors rated all domains independently from these statements, the second assessor being blinded to the first assessor's verdicts. Disagreements were resolved by discussion, with adjudication by a third author where needed. For the 24 trials identified by the original search, the assessors agreed on 115 of 120 domain judgments (95.8%; Cohen's κ 0.92, bootstrap 95% CI 0.84 to 0.98, 5,000 resamples) and on 23 of 24 overall judgments (κ 0.93). The five trials identified during the search update were assessed in the same way.

Overall judgments followed the RoB 2 decision rules: low risk when all five domains were at low risk; high risk when at least one domain was at high risk or when some concerns in multiple domains substantially lowered confidence in the result; and some concerns otherwise. The multiple-domain criterion was applied as some concerns in three or more domains (rated high risk). Trials with some concerns in exactly two domains were judged individually; the reasons are given in Table S5c. D1, randomization process; D2, deviations from intended interventions; D3, missing outcome data; D4, measurement of the outcome; D5, selection of the reported result.

Table S5a. Consensus domain-level and overall RoB 2 judgments.

Trial	D1	D2	D3	D4	D5	Overall	Basis for overall judgment
Abdelaziz 2024	Low	Some concerns	Low	Some concerns	Low	Some concerns	Some concerns in two domains (D2, D4): judged individually, retained at some concerns (Table S5c)
Alli 2026	Low	Low	Low	Low	Low	Low	All domains at low risk
Cameron 2025	Some concerns	Some concerns	Low	Low	Low	Some concerns	Some concerns in two domains (D1, D2): judged individually, retained at some concerns (Table S5c)
Cui 2026	Low	Low	Some concerns	Low	Low	Some concerns	Some concerns in one domain (D3)
Demarquette 2025	Low	Some concerns	Low	Some concerns	Low	Some concerns	Some concerns in two domains (D2, D4): judged individually, retained at some concerns (Table S5c)
Elbardan 2024	Low	Low	Low	Low	Low	Low	All domains at low risk
Eloraby 2024	Low	High	Low	Some concerns	Low	High	High risk in D2
Fadhlurrahman 2024	Some concerns	Some concerns	Low	Some concerns	High	High	High risk in D5
Hamed 2022	Low	Low	Low	Low	Low	Low	All domains at low risk
Huette 2026	Some concerns	Some concerns	Low	Some concerns	Low	High	Some concerns in 3 domains (D1, D2, D4): high risk under the multiple-domain rule
Kalli 2025	Low	Low	Low	Low	Some concerns	Some concerns	Some concerns in one domain (D5)
Kaya 2022	Some concerns	Low	Low	Low	Some concerns	Some concerns	Some concerns in two domains (D1, D5): judged individually, retained at some concerns (Table S5c)
Keshwani 2023	Some concerns	Some concerns	High	Some concerns	Some concerns	High	High risk in D3
Khera 2021	Low	Low	Low	Some concerns	Some concerns	Some concerns	Some concerns in two domains (D4, D5): judged individually, retained at some concerns (Table S5c)
Kumar 2021	Some concerns	Some concerns	Low	Some concerns	Low	High	Some concerns in 3 domains (D1, D2, D4): high risk under the multiple-domain rule
Mahrose 2024	Some concerns	Low	Low	Some concerns	Low	Some concerns	Some concerns in two domains (D1, D4): judged individually, retained at some concerns (Table S5c)
Malik 2026	Low	Some concerns	Some concerns	Some concerns	Some concerns	High	Some concerns in 4 domains (D2, D3, D4, D5): high risk under the multiple-domain rule
Mansour 2024	Some concerns	High	Low	Some concerns	Low	High	High risk in D2
Pascarella 2023	Some concerns	High	Low	Some concerns	Low	High	High risk in D2

Trial	D1	D2	D3	D4	D5	Overall	Basis for overall judgment
Powers 2025	Low	High	Low	High	Low	High	High risk in D2, D4
Shih 2022	Some concerns	Low	Low	Low	Low	Some concerns	Some concerns in one domain (D1)
Singh 2024	Some concerns	Some concerns	Some concerns	Some concerns	Low	High	Some concerns in 4 domains (D1, D2, D3, D4): high risk under the multiple-domain rule
Toker 2025	Low	Some concerns	Some concerns	Some concerns	Low	High	Some concerns in 3 domains (D2, D3, D4): high risk under the multiple-domain rule
Vanjare 2025	Low	Low	Low	Low	Some concerns	Some concerns	Some concerns in one domain (D5)
Wang 2023	Low	Low	Low	Low	Some concerns	Some concerns	Some concerns in one domain (D5)
Wang X 2025	Low	Low	Low	Low	Low	Low	All domains at low risk
Yadav 2024	Low	Some concerns	Low	Some concerns	Low	Some concerns	Some concerns in two domains (D2, D4): judged individually, retained at some concerns (Table S5c)
Zhang 2022	Some concerns	Low	Some concerns	Low	Low	Some concerns	Some concerns in two domains (D1, D3): judged individually, retained at some concerns (Table S5c)
Zhao 2025	Some concerns	High	Low	High	Low	High	High risk in D2, D4

Distribution of overall judgments: low risk 4, some concerns 13, high risk 12 (of 29 trials).

Table S5b. Support for consensus judgments, by domain (judgment in parentheses).

Trial	D1 Randomization process	D2 Deviations from intended interventions	D3 Missing outcome data	D4 Measurement of the outcome	D5 Selection of the reported result
Abdelaziz 2024	(Low) Computer-generated randomization with opaque sealed envelopes; groups comparable at baseline.	(Some concerns) Sham block with 1 mL saline per side, not volume-matched with the active block (20 mL per side); block anesthesiologist aware of allocation; patients under general anesthesia and outcome staff blinded; intraoperative fentanyl titrated to response.	(Low) All 40 randomized patients analyzed.	(Some concerns) Blinded data collector, but blinding may have been compromised by the non-volume-matched sham; opioid consumption recorded over ICU stays of unequal duration.	(Low) Registered (NCT04343105); outcomes reported as registered.
Alli 2026	(Low) Computer-generated sequence (block size 4, stratified by site, sex and surgery type) implemented through a central electronic data capture system.	(Low) Double-blind, placebo-controlled design with blinded study solutions; modified intention-to-treat population excluded 22 of 340 randomized participants in whom the assigned intervention was not started, with similar proportions and reasons in both groups.	(Low) Outcome data available for 318 of 340 randomized participants (94%), with balanced exclusions.	(Low) Opioid consumption abstracted from medication records by outcome assessors blinded to allocation.	(Low) Registered (NCT06028126) with published protocol and statistical analysis plan; primary outcome analyzed as prespecified.
Cameron 2025	(Some concerns) Randomization stratified by sex in blocks of four; method of allocation concealment not reported.	(Some concerns) No sham injection (identical dressings applied); performing anesthetist aware of allocation; patients under general anesthesia and postoperative team, research coordinator and analyst blinded; no unblinding reported.	(Low) 60 of 62 randomized patients analyzed; two exclusions for postoperative pacing requirements.	(Low) Pain and opioid consumption assessed by blinded postoperative staff and from nursing records.	(Low) Registered (NCT05191745); all registered outcomes reported.
Cui 2026	(Low) Computer-generated block randomization by an independent researcher; sequentially numbered sealed opaque envelopes.	(Low) Double-blind factorial design with saline placebo block; patients, surgeons, anesthesiologists, follow-up staff and statisticians blinded.	(Some concerns) 260 of 284 randomized patients (92%) analyzed; losses mainly due to intraoperative conversion to cardiopulmonary bypass, postoperative cerebral infarction or death.	(Low) Opioid consumption expressed as morphine equivalents and recorded by blinded follow-up staff.	(Low) Registered in the Chinese Clinical Trial Registry; outcomes reported.

Trial	D1 Randomization process	D2 Deviations from intended interventions	D3 Missing outcome data	D4 Measurement of the outcome	D5 Selection of the reported result
Demarquette 2025	(Low) Randomized 1:1:1 with stratification on internal thoracic artery harvesting; groups balanced at baseline; protocol published.	(Some concerns) Patients unaware of allocation, but no sham injection; clinicians aware of allocation; all randomized patients analyzed by intention to treat.	(Low) No loss to follow-up; missing QoR-15 data handled by multiple imputation with sensitivity analyses.	(Some concerns) Patient-reported outcomes collected without a sham procedure; blinding of outcome assessors not reported.	(Low) Registered (NCT05345639) with published protocol and prespecified hierarchical testing; outcomes reported as planned.
Elbardan 2024	(Low) Computer-generated randomization; opaque envelopes opened before the block.	(Low) Both groups received an active block; patients and data collectors blinded; blocks performed by an anesthesiologist not involved in data collection.	(Low) All 100 randomized patients analyzed.	(Low) Blinded outcome assessment; fentanyl consumption recorded objectively.	(Low) Registered (NCT05475561); prespecified outcomes reported.
Eloraby 2024	(Low) Allocation using sealed opaque envelopes; groups comparable at baseline.	(High) Control group received no block and no sham procedure despite a double-blind description; single unblinded block operator; blinding of intensive care staff not described.	(Low) All 90 randomized patients followed up and analyzed.	(Some concerns) Outcome assessor unaware of group allocation, but self-reported pain in patients without a sham procedure; duration of mechanical ventilation reported with ambiguous units.	(Low) Registered (NCT05115357); pain and analgesic outcomes reported.
Fadhlurrahman 2024	(Some concerns) Simple randomization by envelope lottery; allocation concealment unclear.	(Some concerns) Described as double-blind, but no sham injection in the control group; blocks performed by different anesthesiologists.	(Low) All 40 randomized patients analyzed.	(Some concerns) Observer blinded, but subjective pain scores in patients without a sham procedure.	(High) No trial registration or protocol; primary outcome was stress markers; unusual data presentation (mean ± $t\alpha/2 \cdot SE$) and selective emphasis of results.
Hamed 2022	(Low) Computer-generated random numbers with opaque envelopes.	(Low) Triple-blind, sham-controlled design (dry needling without injectate); single block operator.	(Low) 70 of 72 randomized patients analyzed; one exclusion per group for surgical re-exploration.	(Low) Blinded outcome assessment; morphine consumption recorded objectively.	(Low) Registered (NCT04134637); prespecified outcomes reported.
Huette 2026	(Some concerns) Randomized 1:1; methods of sequence generation and allocation concealment not described in the report.	(Some concerns) Single-blind design with no sham procedure in the control group.	(Low) Intention-to-treat analysis of 82 patients with multiple imputation for missing data.	(Some concerns) Blinded endpoint evaluation for the primary spirometric outcome, but pain and opioid use recorded without a sham procedure.	(Low) Registered (NCT05515809); primary outcome and correction for multiple testing prespecified.
Kalli 2025	(Low) Block randomization by a research assistant; allocations concealed in sequentially numbered sealed opaque envelopes.	(Low) Saline sham prepared by the anesthesia nurse in unlabeled syringes; surgeon, attending anesthetist and ICU nurses not informed of allocation.	(Low) All 74 randomized patients analyzed.	(Low) Oxycodone consumption recorded from drug administration records under blinded, sham-controlled conditions.	(Some concerns) Registered (NCT03704753), but opioid consumption was a secondary outcome reported per body mass index in the main report; 24-hour consumption reported in a companion report.
Kaya 2022	(Some concerns) Computer-generated randomization with sealed opaque envelopes, but imbalance between groups in sex distribution and ASA physical status.	(Low) Double-blind design in which both groups received an active block; single experienced operator.	(Low) 39 of 42 randomized patients analyzed (two deaths, one redo surgery).	(Low) Blinded outcome assessors; morphine consumption recorded by patient-controlled analgesia.	(Some concerns) Registered (NCT04791592) pilot trial; satisfaction outcome incompletely reported.
Keshwani 2023	(Some concerns) Computer-generated randomization; method of allocation concealment not reported.	(Some concerns) Patients and operators not blinded because the two blocks required different positioning; acute pain service team blinded.	(High) Outcome data for 30 of 38 randomized patients (79%); losses due to blocks not received and loss to follow-up.	(Some concerns) Blinded pain assessment team, but patients aware of the block technique; inconsistent description of the pain scale.	(Some concerns) Registered in the Clinical Trial Registry of India; pain scores at individual time points not reported numerically.
Khera 2021	(Low) Computer-generated random numbers; allocation by the research pharmacy.	(Low) Quadruple-blind, placebo-controlled design with pharmacy-prepared identical solutions.	(Low) All 80 randomized patients followed up and analyzed.	(Some concerns) Pain scores recorded by nursing staff at variable intervals within physician-directed analgesic management.	(Some concerns) Registered (NCT03482973); pain scores at individual time points presented graphically only; opioid consumption reported cumulatively over 48 hours.

Trial	D1 Randomization process	D2 Deviations from intended interventions	D3 Missing outcome data	D4 Measurement of the outcome	D5 Selection of the reported result
Kumar 2021	(Some concerns) Computer-based randomization; method of allocation concealment not described.	(Some concerns) Single-blind design (intensive care nurses); no sham block in the control group; surgical and anesthesia teams aware of allocation.	(Low) All 40 randomized patients analyzed.	(Some concerns) Pain assessed by blinded nurses, but rescue fentanyl given at clinician discretion in patients without a sham procedure.	(Low) Registered (CTRI/2019/08/020729); results consistent with registered outcomes.
Mahrose 2024	(Some concerns) Randomized allocation to two equal groups; methods of sequence generation and allocation concealment not described.	(Low) Double-blind, sham-controlled design (saline block); data collectors, surgeons and intensive care staff blinded; single block operator.	(Low) All 40 randomized patients analyzed.	(Some concerns) Pain scores reported graphically only, and intraoperative fentanyl was titrated by the treating anesthesiologist and differed between groups, which may have influenced postoperative assessments.	(Low) Registered (NCT05774249); outcomes reported as registered.
Malik 2026	(Low) Computer-generated random number table; sealed envelopes opened by a researcher who prepared the study drugs.	(Some concerns) Single-blind design; the control group received no block and no sham procedure.	(Some concerns) 10 of 85 randomized patients (12%) excluded after randomization (surgical re-exploration or extubation after 24 hours); per-protocol analysis.	(Some concerns) Postoperative assessor blinded, but subjective pain scores in patients without a sham procedure.	(Some concerns) Registered (CTRI/2024/02/062531), but the number of randomized patients and some outcome values differ between the abstract, text and tables.
Mansour 2024	(Some concerns) Computer-generated randomization with opaque envelopes, but baseline imbalance in body mass index and ASA physical status.	(High) Not blinded: different ultrasound probes and techniques for the two blocks; blinding of data collectors and intensive care staff not reported.	(Low) All 80 randomized patients analyzed.	(Some concerns) Subjective pain scores with unclear assessor blinding; background fentanyl infusion not quantified by group.	(Low) Registered (NCT05627869); outcomes reported.
Pascarella 2023	(Some concerns) Patients described as freely allocated; methods of sequence generation and allocation concealment not reported; groups comparable at baseline.	(High) No sham procedure; intraoperative fentanyl given at the discretion of unblinded anesthesiologists and markedly different between arms (406 vs 864 μg).	(Low) All 126 patients analyzed.	(Some concerns) No sham procedure and blinding of assessors not confirmed; pain recorded as the maximum score within each interval.	(Low) Registered (NCT04319588); prespecified outcomes reported.
Powers 2025	(Low) Computer-generated random number table in blocks of ten; opaque sealed envelopes opened after induction; groups balanced.	(High) Asymmetric co-interventions: intraoperative morphine infusion in the control arm only and drain-site infiltration in the block arm only; no sham; intraoperative anesthesiologist aware of allocation.	(Low) All 90 randomized patients analyzed.	(High) No sham procedure and unblinded intraoperative anesthesiologist; subjective outcomes; pain scores presented without measures of dispersion.	(Low) Registered (CTRI/2019/10/021591); outcomes reported.
Shih 2022	(Some concerns) Randomization using a password-protected spreadsheet; baseline imbalance in body mass index and diabetes.	(Low) Volume-matched saline sham with identical technique; block performed before the blinded clinical team entered the operating room.	(Low) All nine withdrawals occurred before the intervention; all 52 patients who received the assigned intervention were analyzed.	(Low) Blinded assessors; opioid consumption calculated from medication administration records.	(Low) Registered (NCT04333095); all outcomes reported.
Singh 2024	(Some concerns) Computer-generated randomization; method of allocation concealment not described.	(Some concerns) No sham procedure in the control group; patients and outcome assessor reported as blinded; intraoperative fentanyl standardized.	(Some concerns) 10 of 70 randomized patients (14%) excluded after randomization for hemodynamic instability, unevenly across groups; per-protocol analysis.	(Some concerns) Subjective pain scores in patients without a sham procedure.	(Low) Registered (CTRI/2020/06/025942); outcomes reported with complete data.
Toker 2025	(Low) Computer-generated sequence (1:1:1) implemented by an independent researcher on the day of surgery; groups balanced.	(Some concerns) No sham parasternal injection in the control group; visible puncture sites after the superficial block may have revealed allocation to care providers.	(Some concerns) 75 of 80 randomized patients analyzed (two deaths, two revision surgeries, one postoperative cognitive dysfunction), unevenly across groups.	(Some concerns) Assessor blinded, but pain after extubation is subjective and blinding of intensive care staff may have been compromised.	(Low) Registered (NCT04591119); outcomes reported as planned.

Trial	D1 Randomization process	D2 Deviations from intended interventions	D3 Missing outcome data	D4 Measurement of the outcome	D5 Selection of the reported result
Vanjare 2025	(Low) Computer-generated block randomization with sealed opaque envelopes.	(Low) Both groups received an active block after induction of general anesthesia; the operator was not involved in postoperative observations.	(Low) All 118 randomized patients analyzed.	(Low) Blinded data collector; tramadol consumption recorded objectively.	(Some concerns) Registered (CTRI/2024/01/061291) after enrolment had started (December 2023); pain scores presented graphically only.
Wang 2023	(Low) Computer-generated sequence with variable block sizes; opaque sealed envelopes; groups balanced.	(Low) Saline sham (30 mL per side) with identical technique; patients, anesthesiologists and outcome assessors blinded.	(Low) All 50 randomized patients analyzed.	(Low) Blinded assessment; hydromorphone consumption recorded by patient-controlled analgesia.	(Some concerns) Registered in the Chinese Clinical Trial Registry, but p values for extubation time and intraoperative sufentanil differ between the abstract and the results table, and resting pain scores are presented graphically only.
Wang X 2025	(Low) Computer-generated sequence; sealed opaque envelopes prepared by an independent statistician.	(Low) Double-blind, sham-controlled design with study drug prepared by a separate researcher; intention-to-treat analysis.	(Low) Intention-to-treat analysis of all 110 randomized patients with multiple imputation for missing data.	(Low) Blinded outcome assessors; validated scales; opioid consumption recorded objectively.	(Low) Registered in the Chinese Clinical Trial Registry; prespecified outcomes reported.
Yadav 2024	(Low) Computer-generated sequence (1:1:1); sealed opaque envelopes opened after induction; randomization separate from recruitment.	(Some concerns) No sham block in the control group; performing anesthesiologist aware of allocation; patients under general anesthesia and intensive care team blinded; standardized postoperative analgesia.	(Low) All 60 randomized patients analyzed.	(Some concerns) Subjective pain scores; fentanyl infusion rate titrated within a prescribed range by intensive care staff.	(Low) Registered (CTRI/2021/05/033673); outcomes reported as registered.
Zhang 2022	(Some concerns) Computer-generated randomization; method of allocation concealment not described; timing of exclusions inconsistent between text and flow diagram.	(Low) Sham procedure with saline bolus, catheter and infusion; study solutions prepared by a separate investigator; patients, operators and outcome staff blinded; blinding assessed.	(Some concerns) 6 of 116 enrolled patients (three per group) excluded after allocation (delirium, refusal of blood products, redo surgery); per-protocol analysis.	(Low) Blinded data collector; opioid consumption from patient-controlled analgesia records.	(Low) Registered (ChiCTR2100048244); outcomes reported as registered.
Zhao 2025	(Some concerns) Computer-based randomization; method of allocation concealment not explicitly reported.	(High) Open-label comparison of catheter-based block with intravenous patient-controlled analgesia; no sham procedure; patients and clinicians aware of allocation.	(Low) 112 of 114 randomized patients analyzed (one reoperation, one withdrawal).	(High) Subjective pain scores (visual analogue scale) in patients aware of allocation; assessor blinding did not extend to the comparison with intravenous analgesia.	(Low) Registered in the Chinese Clinical Trial Registry; outcomes reported as specified.

Table S5c. Trials with some concerns in exactly two domains: individual judgment of the overall risk of bias.

Trial	Domains with some concerns	Reason for overall judgment of some concerns
Abdelaziz 2024	D2, D4	The concerns in D2 (non-volume-matched sham) and D4 (possible unblinding of outcome assessment) arise from the same design feature and do not independently add risk; randomization, completeness of data and reporting were at low risk.
Cameron 2025	D1, D2	The D1 concern reflects unreported allocation concealment without baseline imbalance, and the D2 concern the absence of a sham injection, mitigated by blinding of patients, postoperative team and analyst; the two concerns are unrelated and neither was judged to lower confidence substantially.
Demarquette 2025	D2, D4	The concerns in D2 and D4 both arise from the absence of a sham injection in a patient-blinded trial with a published protocol, complete follow-up and prespecified analyses.
Kaya 2022	D1, D5	The D1 concern reflects baseline imbalance in sex and ASA physical status after concealed computer-generated allocation, and the D5 concern incomplete reporting of a secondary outcome not used in this review; both were judged minor.
Khera 2021	D4, D5	The D4 concern relates to the measurement of pain scores and the D5 concern to graphical presentation of individual time points; the quadruple-blind, placebo-controlled design with complete follow-up protects the opioid consumption outcome.
Mahrose 2024	D1, D4	The D1 concern reflects unreported methods of sequence generation and concealment without baseline imbalance, and the D4 concern the graphical reporting of pain and unstandardized intraoperative opioid; the double-blind sham-controlled design with complete follow-up mitigates both.

Trial	Domains with some concerns	Reason for overall judgment of some concerns
Yadav 2024	D2, D4	The concerns in D2 and D4 both arise from the absence of a sham block, mitigated by blinding of the intensive care team and a standardized postoperative analgesic protocol, with concealed allocation and complete follow-up.
Zhang 2022	D1, D3	The D1 concern reflects unreported allocation concealment and the D3 concern a small, balanced number of post-allocation exclusions (3 per group) with documented reasons; the sham-controlled, blinded design was otherwise at low risk.

Table S5d. Differences between the independent ratings and the consensus judgments.

Trial	Domain	Assessor 1	Assessor 2	Consensus	Type	Basis
Kalli 2025	D4	Some concerns	Low	Low	Disagreement	Oxycodone consumption was recorded from drug administration records under blinded, sham-controlled conditions; the concern related to the reporting unit rather than to the measurement of the outcome.
Kumar 2021	D1	Low	Some concerns	Some concerns	Disagreement	Allocation concealment was not described. With some concerns in three domains, the overall judgment was high risk.
Pascarella 2023	D2	Some concerns	High	High	Disagreement	Intraoperative fentanyl was given at the discretion of unblinded anesthesiologists and differed markedly between arms (406 vs 864 μg), a differential co-intervention directly affecting the primary outcome.
Shih 2022	D3	Some concerns	Low	Low	Disagreement	All nine withdrawals occurred before the intervention, and all patients who received the assigned intervention were analyzed.
Zhao 2025	D1	Low	Some concerns	Some concerns	Disagreement	Allocation concealment was not explicitly reported.
Cameron 2025	D1	Low	Low	Some concerns	Consensus decision	The method of allocation concealment was not reported (signalling question 1.2, no information).
Keshwani 2023	D1	Low	Low	Some concerns	Consensus decision	The method of allocation concealment was not reported (signalling question 1.2, no information).
Mahrose 2024	D1	Low	Low	Some concerns	Consensus decision	The methods of sequence generation and allocation concealment were not reported (signalling questions 1.1 and 1.2, no information).

Supplementary Material S6. GRADE evidence profile (Table S6a) and exploratory trial sequential analysis (Table S6b, Figure S1)

Certainty of evidence was assessed with GRADE, starting at high certainty for randomized evidence, and was rated in a non-null effect. Criteria: risk of bias serious if less than 50% of the random-effects weight came from trials at low overall risk of bias and very serious if at least 50% came from trials at high risk; inconsistency serious if $I^2 > 50\%$ and very serious if $I^2 > 75\%$ with trial estimates on both sides of the null; indirectness judged against the population, intervention, comparator, outcome and observation windows; imprecision serious if the 95% CI included no effect or fewer than 400 participants (300 events) contributed, and very serious if both applied or, for odds ratios, if the 95% CI included both 0.75 and 1.25; publication bias (missing evidence) suspected if funnel-plot asymmetry with larger benefits in smaller trials was supported by Egger's test ($p < 0.10$) or by a change in the statistical conclusion under trim-and-fill (assessed for outcomes with at least 10 trials). Minimal clinically important differences were not used in these ratings.

Table S6a. GRADE evidence profile. MD, mean difference; OR, odds ratio; NRS, numeric rating scale; k, number of trials; N, number of participants. Certainty: ⊕⊕⊕⊕ high; ⊕⊕⊕○ moderate; ⊕⊕○○ low; ⊕○○○ very low.

Outcome	k	N	Effect (95% CI)	Risk of bias	Inconsistency	Indirectness	Imprecision	Publication bias	Certainty
24-h opioid consumption	13	902	MD −6.60 mg (−10.92 to −2.27)	Serious (−1): Only 16% of the weight came from trials at low risk of bias (16% from trials at high risk)	Very serious (−2): $I^2 = 98\%$ with trial estimates on both sides of the null (11 favoring and 1 disfavoring USG-PIFB); 95% prediction interval −22.24 to 9.04 mg	Not serious: Population, intervention and comparators matched the review question; two trials contributed cumulative windows other than 0–24 h (Khera 2021, 48 h; Abdelaziz 2024, ICU stay), but restriction to 0–24 h windows gave a similar estimate; opioids converted to intravenous morphine equivalents with standard ratios	Not serious: 95% CI excludes no effect; 902 participants (above the optimal information size of 400)	Suspected (−1): Funnel asymmetry with larger benefits in smaller trials (Egger's test p = 0.11); the statistical conclusion changed under fixed-effect trim-and-fill	⊕○○○ Very low
Pain at rest, extubation	7	536	MD −1.38 (NRS 0–10) (−2.31 to −0.45)	Very serious (−2): 85% of the analysis weight came from trials at high overall risk of bias	Serious (−1): $I^2 = 97\%$; all trial estimates on the same side of the null; 95% prediction interval −4.05 to 1.28 points (NRS 0–10)	Not serious: Population, intervention, comparators and outcome measurement matched the review question	Not serious: 95% CI excludes no effect; 536 participants (above the optimal information size of 400)	Not suspected: Fewer than 10 trials; small-study effects could not be assessed statistically	⊕○○○ Very low
Pain at rest, 6 h	5	306	MD −1.08 (NRS 0–10) (−2.48 to 0.32)	Very serious (−2): 79% of the analysis weight came from trials at high overall risk of bias	Very serious (−2): $I^2 = 93\%$ with trial estimates on both sides of the null (4 favoring and 1 disfavoring USG-PIFB); 95% prediction interval −4.85 to 2.70 points (NRS 0–10)	Not serious: Population, intervention, comparators and outcome measurement matched the review question	Very serious (−2): 95% CI includes no effect and only 306 participants contributed	Not suspected: Fewer than 10 trials; small-study effects could not be assessed statistically	⊕○○○ Very low
Pain at rest, 12 h	7	501	MD −0.84 (NRS 0–10) (−1.39 to −0.28)	Very serious (−2): 83% of the analysis weight came from trials at high overall risk of bias	Serious (−1): $I^2 = 79\%$; all trial estimates on the same side of the null; 95% prediction interval −2.32 to 0.65 points (NRS 0–10)	Not serious: Population, intervention, comparators and outcome measurement matched the review question	Not serious: 95% CI excludes no effect; 501 participants (above the optimal information size of 400)	Not suspected: Fewer than 10 trials; small-study effects could not be assessed statistically	⊕○○○ Very low
Pain at rest, 24 h	12	880	MD −0.90 (NRS 0–10) (−1.54 to −0.26)	Very serious (−2): 61% of the analysis weight came from trials at high overall risk of bias	Very serious (−2): $I^2 = 97\%$ with trial estimates on both sides of the null (10 favoring and 1 disfavoring USG-PIFB); 95% prediction interval −3.10 to 1.31 points (NRS 0–10)	Not serious: Population, intervention, comparators and outcome measurement matched the review question	Not serious: 95% CI excludes no effect; 880 participants (above the optimal information size of 400)	Not suspected: No small-study effects suggesting overestimation (Egger's test p = 0.30)	⊕○○○ Very low

Outcome	k	N	Effect (95% CI)	Risk of bias	Inconsistency	Indirectness	Imprecision	Publication bias	Certainty
Pain during movement, extubation	3	165	MD −0.75 (NRS 0–10) (−1.94 to 0.45)	Very serious (−2): 100% of the analysis weight came from trials at high overall risk of bias	Serious (−1): I^2 = 80%; all trial estimates on the same side of the null; 95% prediction interval −7.18 to 5.69 points (NRS 0–10)	Not serious: Pain during movement assessed during coughing, deep breathing or physiotherapy according to the trial protocols; outcome measure matched the review question	Very serious (−2): 95% CI includes no effect and only 165 participants contributed	Not suspected: Fewer than 10 trials; small-study effects could not be assessed statistically	⨁◯◯◯ Very low
Pain during movement, 6 h	2	80	MD −1.79 (NRS 0–10) (−4.31 to 0.73)	Very serious (−2): 100% of the analysis weight came from trials at high overall risk of bias	Not serious: I^2 = 0%	Not serious: Pain during movement assessed during coughing, deep breathing or physiotherapy according to the trial protocols; outcome measure matched the review question	Very serious (−2): 95% CI includes no effect and only 80 participants contributed	Not suspected: Fewer than 10 trials; small-study effects could not be assessed statistically	⨁◯◯◯ Very low
Pain during movement, 12 h	4	205	MD −1.29 (NRS 0–10) (−1.81 to −0.77)	Very serious (−2): 87% of the analysis weight came from trials at high overall risk of bias	Not serious: I^2 = 0%	Not serious: Pain during movement assessed during coughing, deep breathing or physiotherapy according to the trial protocols; outcome measure matched the review question	Serious (−1): 95% CI excludes no effect but only 205 participants contributed (below the optimal information size of 400)	Not suspected: Fewer than 10 trials; small-study effects could not be assessed statistically	⨁◯◯◯ Very low
Pain during movement, 24 h	6	417	MD −1.01 (NRS 0–10) (−2.05 to 0.04)	Very serious (−2): 69% of the analysis weight came from trials at high overall risk of bias	Very serious (−2): I^2 = 92% with trial estimates on both sides of the null (5 favoring and 1 disfavoring USG-PIFB); 95% prediction interval −3.81 to 1.79 points (NRS 0–10)	Not serious: Pain during movement assessed during coughing, deep breathing or physiotherapy according to the trial protocols; outcome measure matched the review question	Serious (−1): 95% CI includes no effect (417 participants)	Not suspected: Fewer than 10 trials; small-study effects could not be assessed statistically	⨁◯◯◯ Very low
Time to extubation	16	1,563	MD −106.1 min (−174.4 to −37.9)	Serious (−1): Only 20% of the weight came from trials at low risk of bias (46% from trials at high risk)	Very serious (−2): I^2 = 97% with trial estimates on both sides of the null (11 favoring and 4 disfavoring USG-PIFB); 95% prediction interval −374.27 to 162.01 min	Not serious: Population, intervention, comparators and outcome measurement matched the review question	Not serious: 95% CI excludes no effect; 1563 participants (above the optimal information size of 400)	Not suspected: No small-study effects suggesting overestimation (Egger's test p = 0.23)	⨁◯◯◯ Very low
ICU length of stay	14	1,496	MD −13.4 h (−21.3 to −5.4)	Serious (−1): Only 25% of the weight came from trials at low risk of bias (21% from trials at high risk)	Very serious (−2): I^2 = 92% with trial estimates on both sides of the null (12 favoring and 2 disfavoring USG-PIFB); 95% prediction interval −41.85 to 15.14 h	Not serious: Population, intervention, comparators and outcome measurement matched the review question	Not serious: 95% CI excludes no effect; 1496 participants (above the optimal information size of 400)	Not suspected: No small-study effects suggesting overestimation (Egger's test p = 0.68)	⨁◯◯◯ Very low
Hospital length of stay	10	1,116	MD −0.66 days (−1.51 to 0.19)	Serious (−1): Only 37% of the weight came from trials at low risk of bias (19% from trials at high risk)	Very serious (−2): I^2 = 92% with trial estimates on both sides of the null (6 favoring and 4 disfavoring USG-PIFB); 95% prediction interval −3.20 to 1.88 days	Not serious: Population, intervention, comparators and outcome measurement matched the review question	Serious (−1): 95% CI includes no effect (1116 participants)	Not suspected: No small-study effects suggesting overestimation (Egger's test p = 0.22)	⨁◯◯◯ Very low
Rescue analgesic use	6	596	OR 0.61 (0.32 to 1.15)	Serious (−1): Only 0% of the weight came from trials at low risk of bias (25% from trials at high risk)	Not serious: I^2 = 31%	Not serious: Population, intervention, comparators and outcome measurement matched the review question	Serious (−1): 95% CI includes no effect; 199 events (fewer than 300)	Not suspected: Fewer than 10 trials; small-study effects could not be assessed statistically	⨁⨁◯◯ Low
Postoperative nausea and vomiting	7	720	OR 0.62 (0.24 to 1.61)	Serious (−1): Only 20% of the weight came from trials at low risk of bias (30% from trials at high risk)	Serious (−1): I^2 = 75%; 95% prediction interval OR 0.06 to 6.67	Not serious: Population, intervention, comparators and outcome measurement matched the review question	Very serious (−2): 95% CI (OR 0.24 to 1.61) includes both appreciable benefit and appreciable harm; 209 events	Not suspected: Fewer than 10 trials; small-study effects could not be assessed statistically	⨁◯◯◯ Very low

Outcome	k	N	Effect (95% CI)	Risk of bias	Inconsistency	Indirectness	Imprecision	Publication bias	Certainty
USG-PIFB versus deep parasternal techniques, 24-h opioid consumption	6	496	MD 0.43 mg (−3.93 to 4.79)	Serious (−1): Only 0% of the weight came from trials at low risk of bias (41% from trials at high risk)	Very serious (−2): I^2 = 90% with trial estimates on both sides of the null (3 favoring and 3 disfavoring USG-PIFB); 95% prediction interval −8.85 to 9.72 mg	Not serious: Deep parasternal intercostal plane and transversus thoracic muscle plane blocks pooled as one comparator class; all trials compared ultrasound-guided techniques directly	Serious (−1): 95% CI includes no effect (496 participants)	Not suspected: Fewer than 10 trials; small-study effects could not be assessed statistically	⊕○○○ Very low
Local anesthetic systemic toxicity	16	1,215	0/606 vs 0/609 events (USG-PIFB vs comparator)	Serious (−1): Adverse events were not systematically screened for or prespecified in most trials, and 10 of the 29 trials did not report block-related complications	Not serious: No inconsistency between trials	Not serious: Direct evidence from randomized comparisons	Serious (−1): Zero events; the upper 95% confidence limit for the incidence after USG-PIFB is about 0.5% (rule of three)	Not suspected beyond the incomplete reporting already considered under risk of bias	⊕⊕○○ Low
Block-site hematoma	17	1,255	1/626 vs 3/629 events (USG-PIFB vs comparator)	Serious (−1): Adverse events were not systematically screened for or prespecified in most trials, and 10 of the 29 trials did not report block-related complications	Not serious: No inconsistency between trials	Not serious: Direct evidence from randomized comparisons	Serious (−1): Very few events	Not suspected beyond the incomplete reporting already considered under risk of bias	⊕⊕○○ Low
Pneumothorax	17	1,475	8/732 vs 8/743 events (USG-PIFB vs comparator)	Serious (−1): Adverse events were not systematically screened for or prespecified in most trials, and 10 of the 29 trials did not report block-related complications	Not serious: No inconsistency between trials	Not serious: Direct evidence from randomized comparisons	Serious (−1): Very few events	Not suspected beyond the incomplete reporting already considered under risk of bias	⊕⊕○○ Low

Table S6b. Exploratory trial sequential analysis of 24-hour opioid consumption. Two-sided α = 5%, power 90%, variance from the pooled control-group standard deviation (11.2 mg); required information size (RIS) adjusted for diversity (D^2). Lan–DeMets O'Brien–Fleming monitoring boundaries were computed by recursive numerical integration at the cumulative information of each trial (trials ordered by year of publication). No conclusion about the presence or absence of an effect was based on this analysis.

Anticipated MD (mg)	Unadjusted RIS	Diversity (D^2)	Diversity-adjusted RIS	Accrued participants (% of adjusted RIS)	Final cumulative z	Monitoring boundary at final analysis (|z|)	Boundary crossed
5	210	98.8%	17,677	902 (5%)	−3.32	> 8	No
7.5	93	98.8%	7,856	902 (11%)	−3.32	5.80	No
10	53	98.8%	4,419	902 (20%)	−3.32	4.36	No
15	23	98.8%	1,964	902 (46%)	−3.32	2.97	Yes

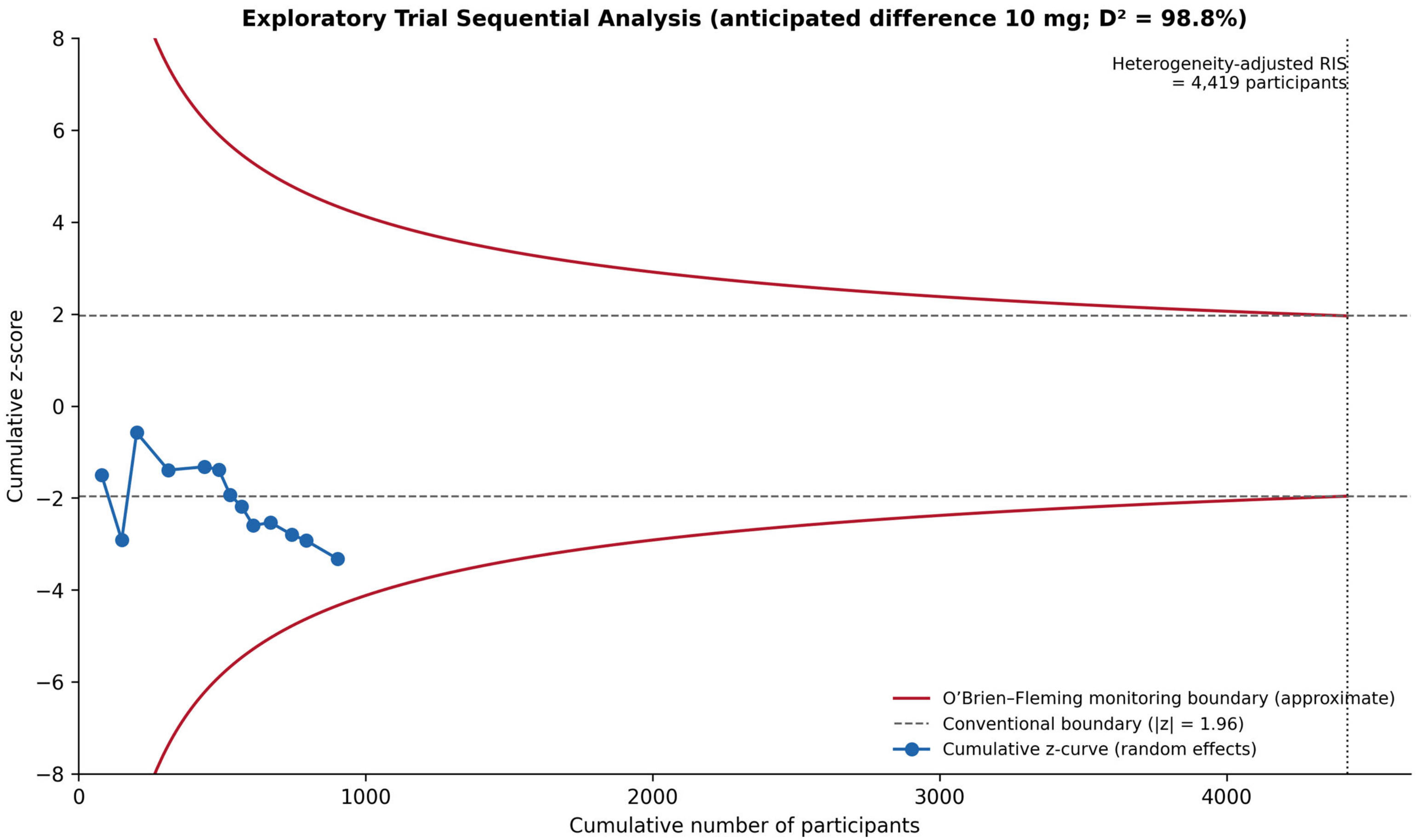

Figure S1. Exploratory trial sequential analysis for an anticipated difference of 10 mg. The cumulative z-curve of the random-effects meta-analysis (trials ordered by year of publication) is shown with the conventional significance boundaries (|z| = 1.96) and the O'Brien–Fleming monitoring boundaries, drawn as a continuous approximation; the boundary values at the actual cumulative information are given in Table S6b. The vertical dotted line marks the diversity-adjusted required information size (4,419 participants).

Supplementary Material S7. Block-related adverse events (Table S7)

Study-level reporting of block-related complications in the 29 included trials. Events are given as USG-PIFB arm/comparator arm (for multi-arm trials, the USG-PIFB and control arms of Comparison 1; for head-to-head trials, the USG-PIFB and alternative-block arms). A general statement that no block-related complications or adverse events had occurred was coded as zero events for each complication type. NR, not reported; LAST, local anesthetic systemic toxicity.

Table S7. Block-related complications by trial.

Trial	Comparison	n (USG-PIFB/comparator)	Pneumothorax	Block-site hematoma	LAST	Details as reported
Abdelaziz 2024	C1	20/20	NR	1/3	NR	Block-site hematoma 1/20 after USG-PIFB vs injection-site hematoma 3/20 after sham injection; pneumothorax and local anesthetic toxicity not reported
Alli 2026	C1	164/154	NR	NR	NR	No serious block-related complications; other block-related events not reported by arm
Cameron 2025	C1	28/32	0/0	0/0	0/0	No pneumothorax or local anesthetic toxicity; no complications detected
Cui 2026	C1	126/134	4/6	NR	NR	Pneumothorax reported as a postoperative complication (4/126 vs 6/134), not attributed to the block; hematoma and local anesthetic toxicity not reported
Demarquette 2025	C1 + C2	84/85	4/2	0/0	0/0	Pneumothorax reported as a postoperative complication in all arms (deep parasternal block arm 10/85), not attributed to the superficial block; no hematoma or puncture-site infection; adverse effects attributed to ropivacaine (2/84 USG-PIFB, 2/85 deep block) were nausea and vomiting and one episode of arterial hypotension (deep block)
Elbardan 2024	C2	50/50	0/0	0/0	0/0	No technique-related or local anesthetic toxicity-related problems
Eloraby 2024	C1	30/30	NR	NR	NR	Hematoma, pneumothorax and local anesthetic toxicity listed as outcomes in the methods but not reported in the results.
Fadhlurrahman 2024	C1	20/20	NR	NR	NR	Block-related complications not reported.
Hamed 2022	C1	35/35	0/0	0/0	0/0	No block-related complications
Huette 2026	C1	41/41	0/0	0/0	0/0	No block-related adverse events
Kalli 2025	C1	37/37	NR	NR	NR	Block-related complications not reported.
Kaya 2022	C2	21/21	0/0	0/0	0/0	No block-related complications
Keshwani 2023	C2	19/19	NR	NR	NR	Block-related complications not reported.
Khera 2021	C1	40/40	0/0	0/0	0/0	No intervention-related adverse events (injection-site hematoma and local anesthetic toxicity monitored)
Kumar 2021	C1	20/20	0/0	0/0	0/0	No complications related to the block (hematoma, wound infection, prolonged paresthesia, pneumothorax)
Mahrose 2024	C1	20/20	0/0	0/0	0/0	No complications related to the block in either group
Malik 2026	C1	50/25	NR	NR	NR	Block-related complications not reported.
Mansour 2024	C2	40/40	NR	NR	NR	Only hemodynamic, sedation and opioid-related side effects reported.
Pascarella 2023	C1	63/63	NR	NR	NR	Only nausea, vomiting and delirium reported.
Powers 2025	C1	45/45	0/0	0/0	0/0	No chest wall hematoma, pneumothorax or vascular injury
Shih 2022	C1	27/25	0/0	0/0	0/0	No adverse events related to the block or placebo procedure
Singh 2024	C1	23/22	0/0	0/0	0/0	No hematoma, pleural puncture or local anesthetic toxicity
Toker 2025	C1 + C2	25/25	0/0	0/0	0/0	No block-related complications
Vanjare 2025	C2	59/59	NR	NR	NR	Only nausea, vomiting and retching reported.
Wang 2023	C1	25/25	NR	NR	NR	Only opioid-related adverse events reported.

Trial	Comparison	n (USG-PIFB/comparator)	Pneumothorax	Block-site hematoma	LAST	Details as reported
Wang X 2025	C1	55/55	0/0	0/0	0/0	No adverse events related to the block (local anesthetic toxicity and puncture-site infection monitored)
Yadav 2024	C1 + C2	20/20	NR	NR	NR	Complications reported graphically as comparable between groups, without specification of block-related events.
Zhang 2022	C1	55/55	0/0	0/0	0/0	No block-related adverse events (hematoma, pneumothorax, ropivacaine allergy, infection)
Zhao 2025	C1	37/38	0/0	0/0	0/0	No adverse reactions attributable to the block or local anesthetic, including local anesthetic toxicity

Summary: 19 of the 29 trials reported block-related complications, at least in part, and 10 did not. No local anesthetic systemic toxicity was reported in the 16 trials reporting this outcome (0/606 USG-PIFB and 0/609 comparator patients; upper limit of the 95% confidence interval for the incidence after USG-PIFB approximately 0.5% by the rule of three). Pneumothorax was reported as a postoperative complication in two trials and was not attributed to the superficial block (8/732 vs 8/743 in the 17 trials reporting this outcome); in the three-arm trial by Demarquette et al., pneumothorax occurred in 10 of 85 patients after the deep parasternal block. One block-site hematoma occurred after USG-PIFB (1/626), and three injection-site hematomas occurred after sham injection (3/629), all in the same trial. No trial was powered for safety outcomes or screened systematically for asymptomatic complications, so these data do not permit a claim of superior safety relative to comparator techniques.
